# The Gut Microbiota Modulates Neuroinflammation in Alzheimer's Disease: Elucidating Crucial Factors and Mechanistic Underpinnings

**DOI:** 10.1111/cns.70091

**Published:** 2024-10-26

**Authors:** Jianshe Yang, Junyi Liang, Niyuan Hu, Ningjuan He, Bin Liu, Guoliang Liu, Ying Qin

**Affiliations:** ^1^ Harbin Institute of Physical Education Harbin Heilongjiang Province China; ^2^ Heilongjiang University of Traditional Chinese Medicine Harbin Heilongjiang Province China

**Keywords:** Alzheimer's disease, dysbiosis, gut microbiota, microbiota–gut–brain axis, microglia, neuroinflammation

## Abstract

**Background and Purpose:**

Alzheimer's disease (AD) is characterized by progressive cognitive decline and neuronal loss, commonly linked to amyloid‐β plaques, neurofibrillary tangles, and neuroinflammation. Recent research highlights the gut microbiota as a key player in modulating neuroinflammation, a critical pathological feature of AD. Understanding the role of the gut microbiota in this process is essential for uncovering new therapeutic avenues and gaining deeper insights into AD pathogenesis.

**Methods:**

This review provides a comprehensive analysis of how gut microbiota influences neuroinflammation and glial cell function in AD. A systematic literature search was conducted, covering studies from 2014 to 2024, including reviews, clinical trials, and animal studies. Keywords such as “gut microbiota,” “Alzheimer's disease,” “neuroinflammation,” and “blood–brain barrier” were used.

**Results:**

Dysbiosis, or the imbalance in gut microbiota composition, has been implicated in the modulation of key AD‐related mechanisms, including neuroinflammation, blood–brain barrier integrity, and neurotransmitter regulation. These disruptions may accelerate the onset and progression of AD. Additionally, therapeutic strategies targeting gut microbiota, such as probiotics, prebiotics, and fecal microbiota transplantation, show promise in modulating AD pathology.

**Conclusions:**

The gut microbiota is a pivotal factor in AD pathogenesis, influencing neuroinflammation and disease progression. Understanding the role of gut microbiota in AD opens avenues for innovative diagnostic, preventive, and therapeutic strategies.

## Graphical abstract

Imbalance in the gut microbiota triggers neuroinflammation, thereby promoting the progression of Alzheimer's disease. Modulating the gut microbiota may reverse this process, offering a new strategy for the treatment of AD. Such interventions through the gut–brain axis pave new avenues for the prevention and treatment of AD. This figure was created with BioRender.com.

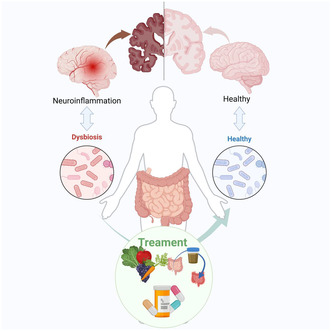

## 1 Introduction

Alzheimer's disease (AD) manifests as a relentless neurodegenerative disorder marked by progressive memory impairment and cognitive deterioration, coupled with the accumulation of amyloid‐beta (Aβ) plaques and neurofibrillary tangles (NFTs) within the brain [1]. Epidemiological investigations indicate a notable surge in AD‐related mortality over the past two decades, cementing its status as the foremost neurodegenerative affliction among the elderly globally [2]. Despite extensive research efforts, the precise pathogenesis of AD remains enigmatic; however, burgeoning evidence implicates neuroinflammation as a central factor in its pathological advancement [3]. Neuroinflammation, predominantly driven by activated neuroglial cells—the innate immune cells of the central nervous system (CNS)—is pivotal in AD [4]. Dysregulation and hyperactivation of neuroglia lead to the excessive secretion of pro‐inflammatory cytokines, reactive oxygen species (ROS), and nitric oxide (NO), thereby exacerbating neuroinflammation and neurotoxicity [4, 5, 6].

The gut microbiota orchestrates cerebral function and influences the onset and progression of AD. It modulates the equilibrium of the gut–brain axis by interacting with the CNS, including the vagus nerve, immune system, and endocrine system [7]. Alterations in the gut microbiota are associated with elevated levels of inflammatory cytokines, oxidative stress, and neurotoxicity in the brains of both AD patients and animal models [8, 9]. The gut microbiota may impact AD by regulating neuroinflammation, as its metabolites may cross the blood–brain barrier (BBB), eliciting anti‐inflammatory and neuroprotective effects on glial cells [10, 11, 12]. Furthermore, the gut microbiota influences the infiltration of peripheral immune cells and the exposure of glial cells to systemic inflammatory stimuli, regulates the expression and activity of pattern recognition receptors, and modulates the production and metabolism of neurotransmitters [13, 14, 15].

This paper aims to provide a comprehensive analysis of the current role of the gut microbiota in modulating neuroinflammation and glial cell function in AD. Its objective is to enhance our understanding of AD's pathogenesis and to deliberate on the potential advantages and challenges of manipulating the gut microbiota as a novel approach for diagnosing, treating, and preventing AD.

## 2 Gut Microbiota and AD

### 2.1 Pathogenesis of AD

**FIGURE 1:**
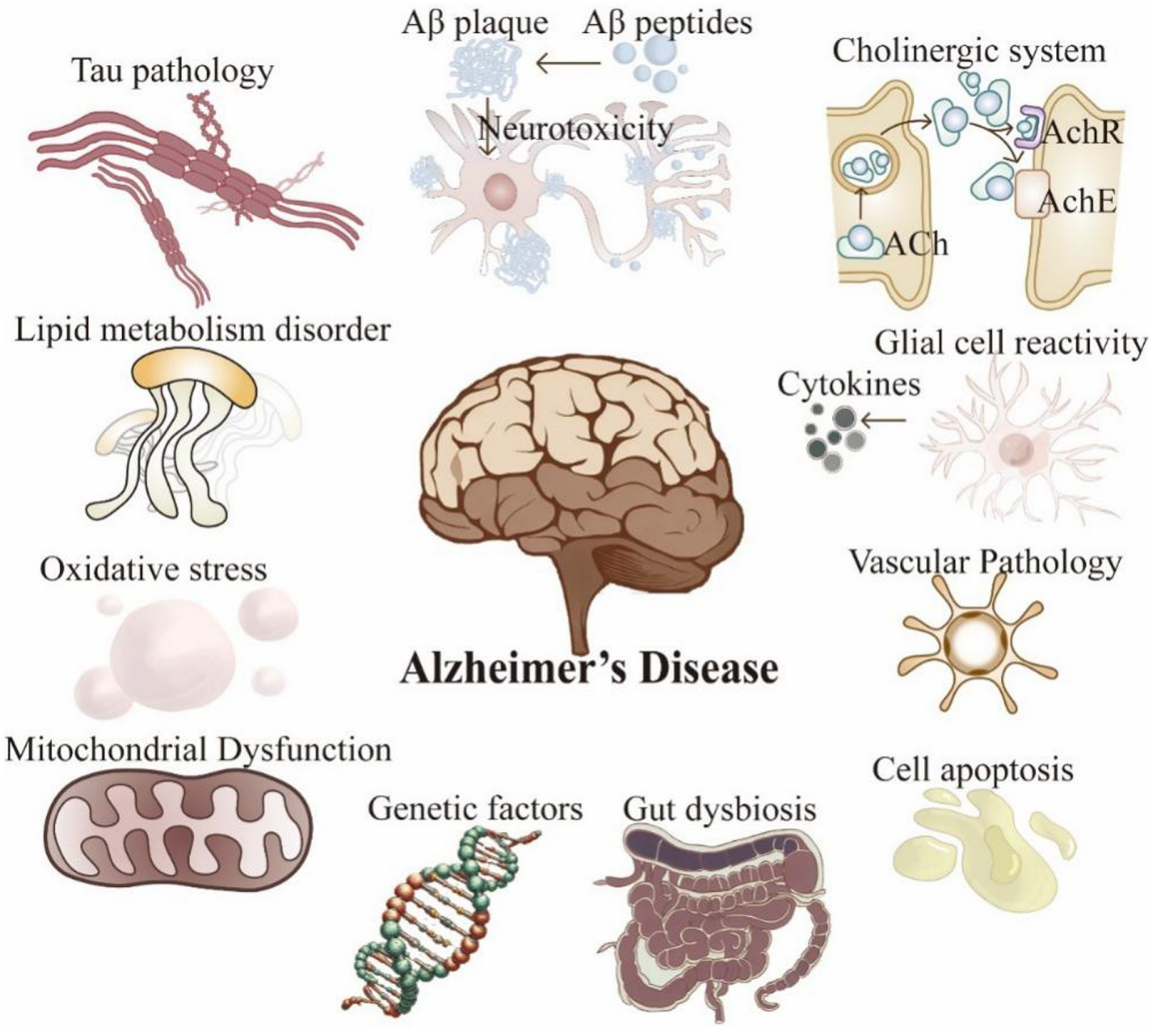
Primary hypotheses concerning the pathogenesis of AD. Risk factors for AD are postulated to correlate with at least one of seven primary pathologies: Apoptosis, oxidative stress, hyperphosphorylation of Tau proteins, Aβ aggregation, pro‐inflammatory cytokines released by reactive glial cells, cerebrovascular pathology, and alterations in the gut microbiota. Each of these primary pathologies may ultimately exacerbate and contribute to the burden of the other major AD pathologies. These pathologies may eventually converge, precipitating insufficient neuronal sustenance, synaptic decay, and demise, culminating in neurodegeneration that clinically manifests as cognitive decline.

Current investigations propose that the etiology and pathogenesis of AD may be influenced by myriad risk factors (Figure 1): (i) The Aβ cascade hypothesis assumes a central role in AD pathology [16]. (ii) The inflammatory response is pivotal in AD pathogenesis, wherein the resultant inflammatory process can precipitate neuronal damage [17, 18]. (iii) The integrity of the BBB is crucial in AD progression, and its compromised state incites inflammatory and neurodegenerative processes [19, 20]. (iv) Other pathological mechanisms [3, 21]: Apart from the aforementioned factors, various elements such as tau protein modifications, the autophagy–lysosomal pathway, mitochondrial function, cholinergic transmission, oxidative stress, and genetic susceptibility may influence AD progression. Notably, disruptions in the gut microbiota may serve as significant factors in AD pathogenesis, influencing Aβ deposition and neuroinflammation [22]. These interwoven factors collectively contribute to AD progression.

### 2.2 Gut Microbiota and the Brain

High‐throughput sequencing technologies have provided us with a deeper understanding of the diversity and abundance of human gut microbes. The magnitude and composition of these microbiota are shaped by various factors, including gender, age, diet, and geography [23]. Gut microbes not only colonize the body's surfaces and fluids but predominantly inhabit the digestive tract [24]. They form an extensive chemical factory that impacts host health through the synthesis of diverse compounds. The gut microbiome plays a pivotal role in modulating host immunity, digestion, and neural signaling and its association with brain function, potentially offering novel insights into the exploration, comprehension, and treatment of neurodegenerative conditions like AD [25]. The microbial–gut–brain axis represents a complex network of connections between the gut and the brain through the nervous, endocrine, and immune systems via multiple pathways (e.g., metabolite pathways, immune response pathways, neurotransmission pathways, neuroendocrine–hypothalamic axis pathways, and gut and BBB), which may act autonomously or synergistically to influence the onset and progression of AD [22, 26].

### 2.3 Alterations in the Gut Microbiome in AD

Recent studies have revealed significant discrepancies in the gut microbiota among individuals suffering from AD [27, 28, 29, 30, 31, 32, 33, 34, 35, 36] or analogous animal models [37, 38, 39, 40, 41, 42, 43, 44, 45, 46], juxtaposed with those of robust health (Table 1). These investigations propose that the alterations noted in the gut microbiome, encompassing reduced microbial diversity, abnormal proliferation or depletion of particular bacterial groups, decreased abundance of probiotics, and variances in microbial metabolites, could represent a pivotal aspect in the pathogenesis of AD [47, 48, 49, 50, 51].

**TABLE 1:** Metamorphosis of gut microbiota in AD context.

Category	Methodology	Major findings	Refs.
AD patients	16S rRNA sequencing	Bacterial population in the brain ↑	[27]
	16S rRNA sequencing	Firmicutes and Bifidobacterium ↓, Bacteroidetes↑	[28]
	qPCR	Escherichia/Shigella ↑ and E. rectale ↓	[29]
	Metabolic phenotyping	Metabolite concentrations of tryptophan pathway metabolites in urine and serum ↓	[30]
	PCR for DNA and DNA sequencing	LPS and gram‐negative *Escherichia coli* fragments colocalize with amyloid plaque	[31]
	16S rRNA sequencing	Various changes in microbial populations associated with amyloid positivity and p‐tau status	[32]
	Behavioral assessment	FMT from healthy donors showed improvements in MMSE score, memory, cognition, mood, and socialization	[35]
MCI patients	16S rRNA sequencing	The abundance of the genus Ruminococcus, Butyricimonas, and Oxalobacter ↓	[36]
	GC–MS and PET	Fecal levels of acetic acid, butyric acid, and caproic acid ↓; Fecal SCFAs in MCI group were negatively associated with Aβ deposition in cognition‐related regions	[50]
	CSF biomarker quantification	TMAO levels in CSF ↑	[51]
Adults (4 Age Groups)	qPCR	Bifidobacterium, Faecalibacterium, Bacteroides group, and Clostridium cluster XIVa ↓ with age up to 66–80 years	[33]
Animal models			
GF mice	RNA‐seq	GF animals display global defects in microglia	[37]
APP/PS1 transgenic mice	16S rRNA sequencing and ^1^H nuclear magnetic resonance	*Proteobacteria* and *Verrucomicrobia* ↑, *Bacteroidetes* and key metabolic components of SCFAs ↓	[38]
Thy1‐C/EBPβ transgenic mice	Immunofluorescent staining and ELISA assays	FMT from AD donors induces Aβ and Tau aggregation, associated with upregulation of the C/EBPβ/AEP pathway, microglia activation, and cognitive impairment	[46]
APP/PS1 transgenic mice	16S rRNA sequencing	Microbiota composition and diversity perturbed	[39]
5xFAD mice	16S rRNA sequencing and Immunofluorescent staining	Dysbiosis of gut flora (Aβ in gut; trypsin ↓; *Firmicutes: Bacteroidetes ratio* ↑; *Clostridium leptum* ↑); C/EBPβ/AEP pathway active in gut with age	[40, 41]
3xTg mice	Immunofluorescent staining	Microbiota accelerates AD pathology with active C/EBPβ/AEP signaling in brain	[41]
3xTg mice	16S rRNA sequencing and Immunostaining	FMT from SPF mice and AD patients to GF 3xTg mice activates C/EBPβ/AEP signaling, promoting microglial activation and cognitive deficits	[42]
P301L mice	16S rRNA sequencing	Firmicutes: Bacteroidetes ratio↓	[43]
5xFAD mice	Amyloid quantification and behavioral assessment	7‐day FMT regimen improved the “plaque‐busting” and cognitive behavior shown in 5xFAD mice	[44]
APP/PS1 transgenic mice and WT mice	ELISA assays and behavioral assessment	FMT from AD donors worsened behavior and increased neuroinflammation; FMT from AD donors to WT increased neuroinflammation	[45]
APP/PS1 transgenic mice	ELISA assays and behavioral assessment	FMT from WT donors improved cognitive function, decreased Aβ plaque burden, and decreased levels of soluble Aβ40 and Aβ42	[38]

*Note:* Arrows: ↑: Increase or higher levels;↓: Decrease or lower levels.

Abbreviations: Aβ, amyloid‐beta; AD, Alzheimer's Disease; AEP, aspartic endopeptidase; C/EBPβ, CCAAT/enhancer‐binding protein beta; CSF, cerebrospinal fluid; FMT, fecal microbiota transplantation; GC–MS, gas chromatography–mass spectrometry; GF, germ‐free; LPS, lipopolysaccharide; MCI, mild cognitive impairment; MMSE, mini‐mental state examination; NMR, nuclear magnetic resonance; PCR, polymerase chain reaction; PET, positron emission tomography; qPCR, quantitative polymerase chain reaction; SPF, specific pathogen‐free.

## 3 Gut Microbiota and Neuroinflammation

Neuroinflammation is the immune response elicited by glial cells within the CNS, typically in reaction to stimuli such as neural injury, infections, toxins, or autoimmunity [52]. In AD, neuroinflammation constitutes the third primary pathological hallmark following Aβ accumulation and NFT formation [53]. Among the principal glial cell types, microglia are distinguished as the predominant innate immune cells of the CNS, serving as the primary responders to Aβ plaques by aggregating in their vicinity [54, 55]. Numerous genome‐wide association studies have further identified microglia as the principal cell type expressing AD‐related genes [56]. Microglia play a pivotal role in detecting and reacting to environmental changes, eliminating harmful stimuli, and presenting antigens to T lymphocytes, ultimately contributing to neurodegeneration [18]. Astrocytes are integral in maintaining the integrity and metabolic coupling of the BBB, regulating ion and neurotransmitter homeostasis, producing neurotrophic factors, and supporting neuronal activity and synaptic function [6]. Under normal circumstances, microglia and astrocytes perform neuroprotective functions, limiting the extent of neuroinflammation. However, in pathological conditions such as AD, the chronic activation of microglia and astrocytes becomes dysregulated and overactivated, leading to sustained low‐grade neuroinflammation and excessive production of pro‐inflammatory cytokines, ROS, and NO, which can be detrimental to neurons and synapses [4]. Indeed, neuroinflammation and the activation of glial cells are hallmark features of AD [57].

Neuroinflammation plays a key role in the pathogenic mechanisms of AD, influencing the metabolism of Aβ and tau, the functionality of neurons and synapses, and accelerating the progression and deterioration of AD. It augments the production and deposition of Aβ, a hydrophobic peptide that, if not cleared, aggregates into neurotoxic oligomers and fibrils, leading to neuronal death and synaptic dysfunction [58]. Neuroinflammation activates β‐secretase and γ‐secretase pathways, enhancing Aβ production while releasing oxidative stress factors, metalloproteinases, and inflammatory cytokines that impair Aβ degradation and clearance [59]. Additionally, neuroinflammation disrupts Aβ transport and elimination, causing its accumulation at the blood–brain barrier. It also catalyzes the aberrant phosphorylation and aggregation of tau [60]. In AD pathology, aberrant tau phosphorylation leads to its detachment from microtubules, culminating in the formation of NFTs and disrupting neuronal metabolism and signal transduction. The activation of various kinases, such as GSK‐3β, CDK5, MAPK, and PKA, induced by neuroinflammation, elevates tau phosphorylation levels [54]. Moreover, the secretion of inflammatory cytokines like tumor necrosis factor‐α (TNF‐α), interleukin‐1β (IL‐1β), and IL‐6 during neuroinflammation exacerbates tau aggregation and dissemination [18]. Neuroinflammation inflicts damage on neurons and synapses, both directly and indirectly, by inducing apoptosis and necrosis through the release of oxidative stress factors, inflammatory cytokines, nitric oxide, and glutamate [4]. Additionally, neuroinflammation disrupts the synthesis, release, and reuptake of neurotransmitters, precipitating synaptic dysfunction and degeneration. The altered expression and signaling pathways of neurotrophic factors further impair neuronal survival and plasticity [61]. Hence, mitigating neuroinflammation emerges as a promising strategy for the prevention and treatment of AD.

**FIGURE 2:**
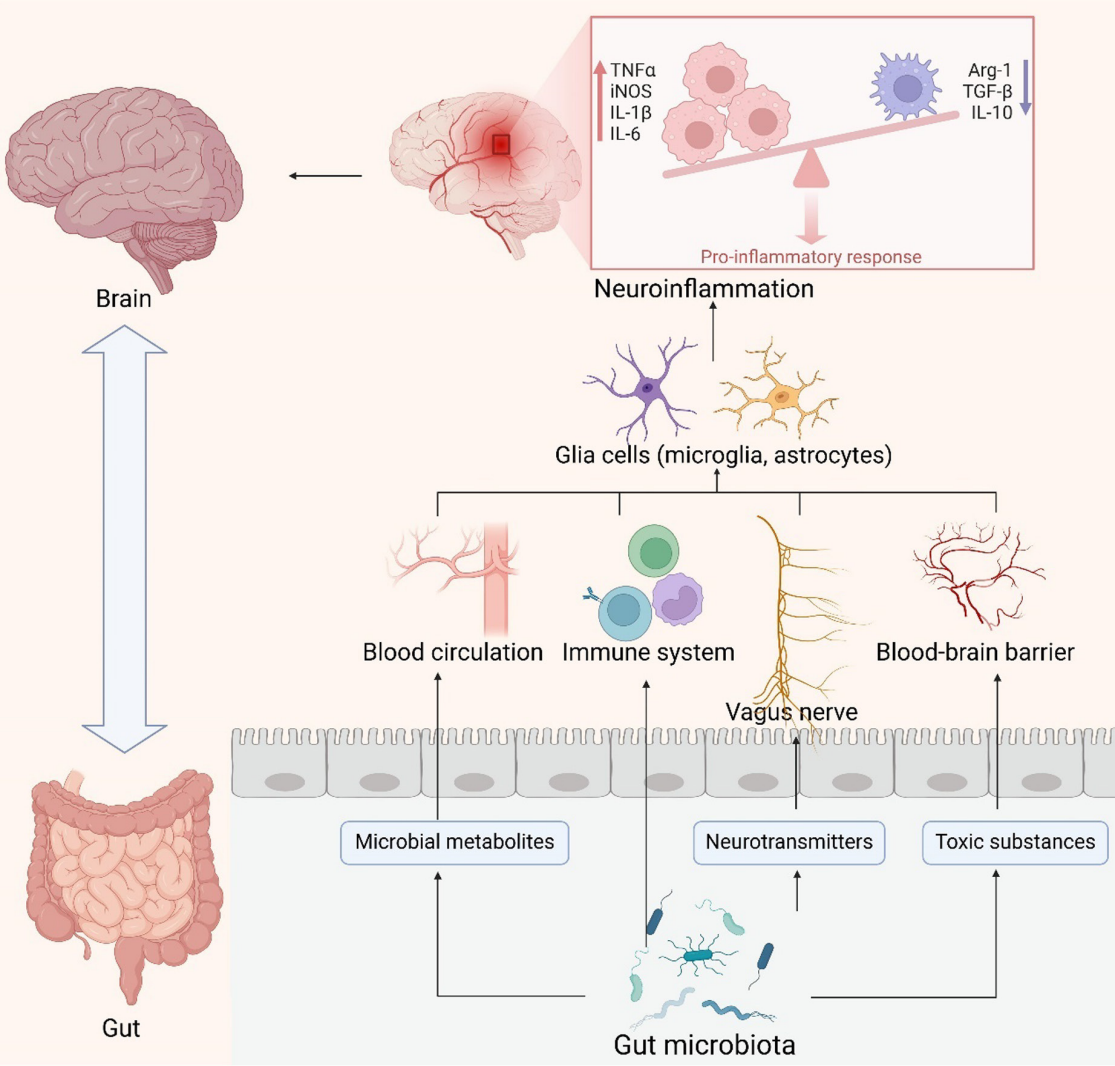
Neuroinflammatory signaling cascades propagated through the intricate network of the gut–brain axis. Bidirectional transmission of inflammatory signals between the intestinal tract and the central nervous system occurs via four predominant avenues: (I) Microbial metabolic pathways; (ii) Immune regulatory pathways; (iii) Neurologic circuits of the Vagus Nerve; and (iv) Induction of compromise in the blood–brain barrier. This figure was created with BioRender.com. Ach, acetylcholine; Kyn, kynurenine; LPS, lipopolysaccharide; SAA, Serum amyloid A; SCFAs, short‐chain fatty acids; TMAO, trimethylamine N‐oxide; Trp, tryptophan; VN, vagus nerve; 5‐HT, 5‐hydroxytryptamine.

Furthermore, the gut microbiota serves as a significant source of amyloid proteins. Bacterial strains such as *Escherichia coli*, *Bacteroides fragilis*, *Salmonella typhi*, *Pseudomonas fluorescens*, and *Staphylococcus aureus* are known to produce amyloid proteins. These strains synthesize proteins like curli, TasA, CsgA, FapC, and phenol‐soluble modulators, which facilitate the misfolding of Aβ fibrils and oligomers. This aberration may compromise the host's immune system [62]. Additionally, these bacteria can produce endotoxins such as lipopolysaccharides (LPS), whose levels rise following bacterial infection or alterations in gut microbial metabolic activity due to inflammation, potentially exacerbating neurodegeneration [63]. Notably, this process may impair brain cell function, initiating a vicious cycle between the gut and the brain [64, 65]. Presently, the comprehension of the mechanisms by which the gut microbiota modulates neuroinflammatory processes in AD remains in its nascent stages. Nonetheless, preliminary evidence from extant research indicates that the gut microbiota exerts a substantial modulatory influence on neuroinflammation via four discernible pathways: the metabolite pathways of the gut microbiota, immune response pathways, neural transmission pathways, and the gut–brain barrier (Figure 2). These pathways, either independently or synergistically, impact the onset and progression of AD. Continued research endeavors hold the promise of enhancing the understanding of the intricate and interrelated interactions between the gut microbiota and AD, thereby offering a novel vantage point for the development of innovative therapeutic strategies.

### 3.1 The Metabolite Pathway

**FIGURE 3:**
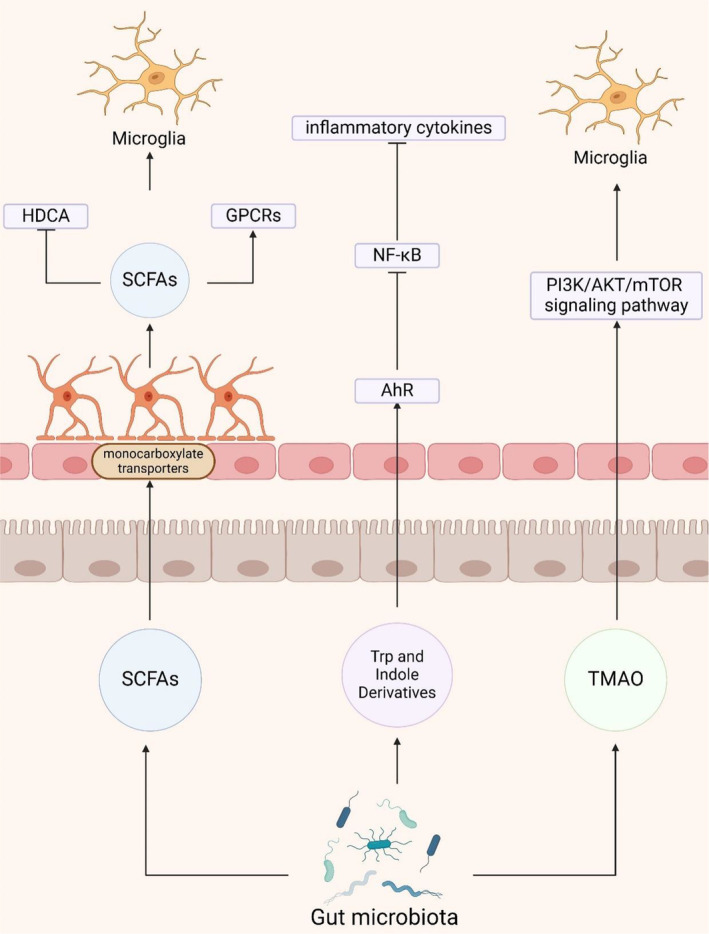
Metabolic pathways of the gut microbiota in AD. Key metabolites produced by the gut microbiota, such as SCFAs, Trp and indole derivatives, and TMAO, influence microglial activity via specific signaling pathways and receptors. This figure was created with BioRender.com. AhR, Aryl Hydrocarbon Receptor; GPCRs, G‐Protein‐Coupled Receptors; HDCA, Hydroxy‐3‐oxo‐4,6,8,11,13‐icosapentaenoic acid; NF‐κB, Nuclear Factor kappa‐light‐chain‐enhancer of activated B cells; PI3K/AKT/mTOR, Phosphoinositide 3‐Kinase/Protein Kinase B/Mechanistic Target of Rapamycin; SCFAs, Short‐Chain Fatty Acids; TMAO, Trimethylamine N‐oxide; Trp, Tryptophan.

The gut microbiota plays an important role in the function and behavior of the neuroinflammatory system, affecting it through the direct or indirect production of metabolites such as short‐chain fatty acids (SCFAs), amino acids, lipopolysaccharides (LPS), and trimethylamine (Figure 3) [66].

#### 3.1.1 Short‐Chain Fatty Acids

SCFAs, primarily composed of butyrate, propionate, and acetate, are fermentation by‐products of the gut microbiota, predominantly derived from the phylum Bacteroidetes and indigestible carbohydrates such as dietary fibers [67]. These SCFAs permeate the BBB via monocarboxylate transporters on endothelial cells and contribute to maintaining BBB integrity by suppressing pathways associated with non‐specific inflammatory responses to microbial infections in vitro [68, 69]. Studies have demonstrated that SCFA mixtures restore the expression levels of claudin‐5, occludin, and ZO‐1 in the hippocampus, thereby improving BBB permeability [70, 71]. Similarly, using hCMEC/D3 cells as an in vitro model of the human BBB, it was observed that propionate mitigated the LPS‐induced disruption of occludin, claudin‐5, and ZO‐1 localization within cells [69]. Thus, dysbiosis of the gut microbiota may compromise the protective role of SCFAs on the BBB, leading to increased permeability and the infiltration of peripheral inflammatory factors into the brain, ultimately contributing to neuroinflammation [72, 73].

SCFAs exert their physiological effects by acting as endogenous ligands for G‐protein‐coupled receptors (GPCRs), particularly GPR43 and GPR41, and by modulating gene expression through inhibition of histone deacetylase (HDAC) activity [12]. In vitro studies have demonstrated that acetate displays anti‐inflammatory properties in Aβ‐induced BV‐2 microglia by enhancing GPR41 expression and inhibiting the ERK/JNK/NF‐κB signaling pathway [74]. Furthermore, in vitro experiments have indicated that SCFAs can diminish histone deacetylase activity and hinder NF‐κB nuclear translocation, thereby directly modulating LPS‐induced primary microglial cells [75]. Animal studies have shown that genetic deletion of microglial Hdac1 and Hdac2 significantly ameliorates cognitive deficits in 5xFAD mice by enhancing microglial Aβ phagocytosis [76]. Meanwhile, augmenting butyrate levels through *Clostridium butyricum* intervention has demonstrated its capacity to suppress microglial activation and reduce pro‐inflammatory cytokine levels in APP/PS1 mice [77]. SCFAs directly interact with microglia, attenuating their antigen‐capturing capabilities and consequently reducing the production of pro‐inflammatory cytokines such as IL‐12 and TNF‐α [78, 79]. These cytokines play a crucial role in regulating the neuroinflammatory response and Aβ deposition in the brain [75]. Despite numerous studies highlighting the pivotal role of SCFAs in mediating communication between gut microbiota and glial cells, a more exhaustive understanding of the mechanisms underlying SCFA actions in AD, including their effects on other neural cells within the brain, necessitates further meticulous exploration.

#### 3.1.2 Tryptophan and Indole Derivatives

Amino acids, as precursors of bioactive molecules, play a pivotal role, with mounting evidence highlighting the critical involvement of the gut microbiota in the metabolic processing and utilization of essential amino acids, particularly tryptophan (Trp) [80]. The majority of dietary L‐tryptophan released in the gut is transferred into circulation via epithelial transport; approximately 10%–20% of L‐tryptophan is metabolized by intestinal epithelial cells and the gut microbiota within the intestinal lumen [81]. The gut microbiota can degrade dietary L‐tryptophan into bioactive metabolites, namely (i) indoles, (ii) kynurenine (Kyn), (iii) serotonin, and (iv) tryptamine pathways [82, 83, 84, 85]. Enterochromaffin cells in the gut are capable of converting dietary L‐tryptophan into serotonin, while the gut microbiota can also modulate the synthesis and release of serotonin from enterochromaffin cells [86]. For instance, Reigstad et al. [87] demonstrated that SCFAs produced by the microbiota promote serotonin production in human enterochromaffin cells. Tryptophan hydroxylase 1 (TPH1), the rate‐limiting enzyme in serotonin biosynthesis, catalyzes the conversion of L‐tryptophan into 5‐hydroxytryptophan, which is subsequently decarboxylated to produce 5‐hydroxytryptamine (5‐HT), or serotonin. Consequently, 5‐HT can be further metabolized into melatonin, regulating various features of the gut microbiota, including oxidative stress and inflammation [88].

Gut microorganism‐mediated Trp metabolism encompasses pathways such as the aryl hydrocarbon receptor (AhR) ligand pathway, the indole pathway, the Kyn pathway, and the 5‐hydroxytryptophan pathway [89]. Metabolites of Trp derived from the gut, such as indole or bacterial tryptophanase, influence astrocytes and microglia via AhR signaling, thereby significantly impacting neuroinflammation in experimental autoimmune encephalomyelitis mice [90, 91]. Additionally, indole metabolites from the gut microbiota activate AhR, inhibit the NF‐κB pathway, suppress the formation of NLRP3 inflammasomes, and reduce the production of inflammatory cytokines, thereby improving gastrointestinal function, modulating microglial reactivity, and alleviating neuroinflammation in APP/PS1 mice [92, 93]. Kyn can traverse the BBB to exert its effects within the brain [94]. Furthermore, Kyn treatment has been shown to upregulate the expression of NLRP2 inflammasomes in astrocytes, resulting in the secretion of IL‐1β and IL‐18 [95]. Although the neuroinflammatory role of gut‐mediated Trp metabolites in AD has been explored to some extent, further research is necessary to elucidate the precise mechanisms underlying the actions of different tryptophan metabolites in AD.

#### 3.1.3 Trimethylamine N‐Oxide

Trimethylamine (TMA) is synthesized by the gut microbiota during the metabolism of methylamine‐containing dietary nutrients [96]. This compound subsequently undergoes hepatic metabolism to form trimethylamine N‐oxide (TMAO) via flavin monooxygenase. TMAO crosses the blood–brain barrier, triggering neurodegeneration by activating microglia and astrocytes and enhancing the release of inflammatory mediators [97, 98]. TMAO promotes inflammation and worsens Aβ and tau pathology in D‐galactose/AlCl3‐induced AD mice through the PI3K/AKT/mTOR signaling pathway [99]. Conversely, administration of the TMA formation inhibitor 3,3‐dimethyl‐1‐butanol diminishes circulating TMAO levels and improves cognitive deficits in APP/PS1 mice by attenuating Aβ pathology and neuroinflammation [100].

### 3.2 The Immune Pathway

The immune and CNS are intricate networks that regulate various physiological functions in the organism [101]. They exhibit shared characteristics in their function and development, potentially contributing to the pathogenesis of neuropsychiatric disorders. Around 70%–80% of immune cells in the human body reside within the gastrointestinal tract, facilitating direct interactions between gut and immune cells [102]. Microbe‐associated molecular patterns produced by pathogenic microbes engage pattern recognition receptors (e.g., TLRs) on host cell surfaces, modulating the production of both pro‐ and anti‐inflammatory cytokines [103]. These cytokines cross the BBB and influence CNS cells, including microglia, thereby shaping the brain's inflammatory environment [104]. The resultant chronic inflammation significantly impacts neurodegeneration [104].

#### 3.2.1 Immune Regulation

Dysregulation of the pro‐inflammatory gut microbiota in AD patients may initiate inflammation and promote the formation and aggregation of Aβ proteins [101]. Accumulation of Aβ in the brain triggers intracerebral immune‐inflammatory responses via TLRs and CD14, predominantly mediated by microglial cells. This cascade results in the release of various cytokines and the upregulation of antigenic markers, precipitating a neuroinflammatory response [105]. This acute and transient inflammation supports Aβ clearance and neuronal protection [105]. Systemic inflammation resulting from intestinal dysregulation may exacerbate microglial hyperactivation and impair hippocampal plasticity, worsening the onset and progression of AD [106]. Disruption of intestinal barrier (IB) function increases permeability to commensal microbes, microbial‐derived products (e.g., metabolites, virulence factors), and other intestinal constituents, leading to aberrant immune‐inflammatory responses such as inflammation, allergies, and autoimmune diseases mediated by molecular mimicry and dysregulated T‐cell responses [66]. T cells play a crucial role in systemic and mucosal immune responses, initiated by dendritic cells continually sampling the intestinal lumen. This process primarily contributes to the proliferation of regulatory T cells (Treg) [107]. Furthermore, the altered composition of gut microbes and their derived metabolites may stimulate or inhibit the differentiation of initial CD4+ T cells into TH17 cells, which are highly abundant at the mucosal barrier and play a key role in regulating tissue homeostasis. Specific intestinal bacteria induce distinct T‐cell subsets; for example, segmented filamentous bacteria drive the differentiation of Th17 cells, while fragile Bacteroides generate Treg expressing the transcription factor Foxp3 [108, 109]. Some commensal microorganisms, such as *Bacteroides fragilis, Bifidobacterium infantis,* and *Firmicutes*, as well as certain microbial metabolites, can influence immune responses by affecting different cell types in the immune environment [106]. They induce the differentiation of Treg into effector cells, such as Th1 and Th17 cells, or IL‐10 regulatory T cells, promoting either immune activation or tolerance [110]. They induce the differentiation of Treg into effector cells, such as Th1 and Th17 cells, or IL‐10 regulatory T cells, promoting either immune activation or tolerance [111, 112, 113, 114, 115]. *Bacillus*‐derived poly‐γ‐glutamic acid specifically signals CD4+ T cells, facilitating selective Treg differentiation [116]. Increased microbiota‐induced Th17 differentiation has been linked to behavioral abnormalities from maternal immune activation, potentially migrating from intestines to meninges [107, 117, 118, 119]. Intestinal microbe–immune cell interactions regulate T‐cell and B‐cell dynamics, impacting immune responses in both peripheral and CNS. Gut T and B cell subsets may migrate from gut to meninges, influencing local neuroimmune environments through cytokine production such as IL‐17a, IL‐10, and IgA antibodies, thereby modulating neuroinflammation [120]. Additionally, microbiota‐derived metabolic products like taurocholic acid, histamine, indole, and spermine influence downstream neuropeptides, regulating NLRP6 inflammasomes, IL‐10, and IL‐18 secretion, which correlate with inflammatory factor levels and Alzheimer's severity [14, 121].

#### 3.2.2 5‐Hydroxytryptamine

Serotonin, also known as 5‐hydroxytryptamine (5‐HT), acts as a neurotransmitter with multifaceted roles in the brain and gut, particularly in orchestrating the gut microbiota‐brain axis [122]. The gut microbiota influences systemic immune function through modulation of 5‐HT production and release from gut enterochromaffin cells [122, 123]. 5‐HT impacts the functionality of monocytes and macrophages, governing inflammatory responses and potentially influencing neuroinflammation [124]. Research indicates that 5‐HT regulates neuroinflammation by activating the 5HT2AR/cAMP/PKA/CREB/Sirt1 pathway and the NF‐κB pathway, controlling the transcription of TLR2 and TLR4 in response to microglial phagocytic stimuli and thereby influencing neuroinflammation [125, 126, 127]. Additionally, 5‐HT modulates the release of inflammatory cytokines, affecting the activation of immune cells and inflammatory responses, such as TNF‐α, IFNγ, IL‐1β, IL‐17, and IL‐6 [128]. Moreover, 5‐HT binding to its receptors on microglia triggers the release of cytokine‐laden exosomes, providing an alternative mechanism for the modulation of gut‐induced neuroinflammation [129]. The synthesis, metabolism, or transport of 5‐HT, critical in the inflammatory response, may offer novel avenues for mitigating neuroinflammation in AD.

#### 3.2.3 Serum Amyloid A

Serum amyloid A (SAA), a product of the inflammatory cascade in intestinal epithelial cells, serves as a prominent acute‐phase reactant, with the gut microbiota potentially modulating neuroinflammation through the regulation of SAA levels [130, 131]. In the brain tissues of AD patients, SAA localizes with the distribution of senile plaques [132]. A recent study reported that SAA expression in the brains of APP/PS1 mice exacerbates neuroinflammation by hindering astrocyte activation and migration toward Aβ plaques via the p38 MAPK pathway [133]. In vitro, recombinant SAA treatment modulates the functions of astrocytes and microglia, decreasing astrocyte viability while enhancing microglial activity [132]. There are also differences in cytokines and inducible iNOS between the two cell types [132]. PI3K is a common pathway mediating the effects of SAA on astrocytes and microglia, whereas the c‐JNK pathway is selectively induced in microglia, and the NF‐kB pathway is selectively activated in astrocytes [132]. Furthermore, SAA promotes the differentiation of Th17 cells, increasing the expression of the pro‐inflammatory cytokine IL‐17, which induces the production of cytokines such as IL‐1β, IL‐6, TNF‐α, and IL‐22 [131, 134]. SAA orchestrates neuroinflammation, regulates cholesterol metabolism, and activates glial cells, impacting AD [135].

**FIGURE 4:**
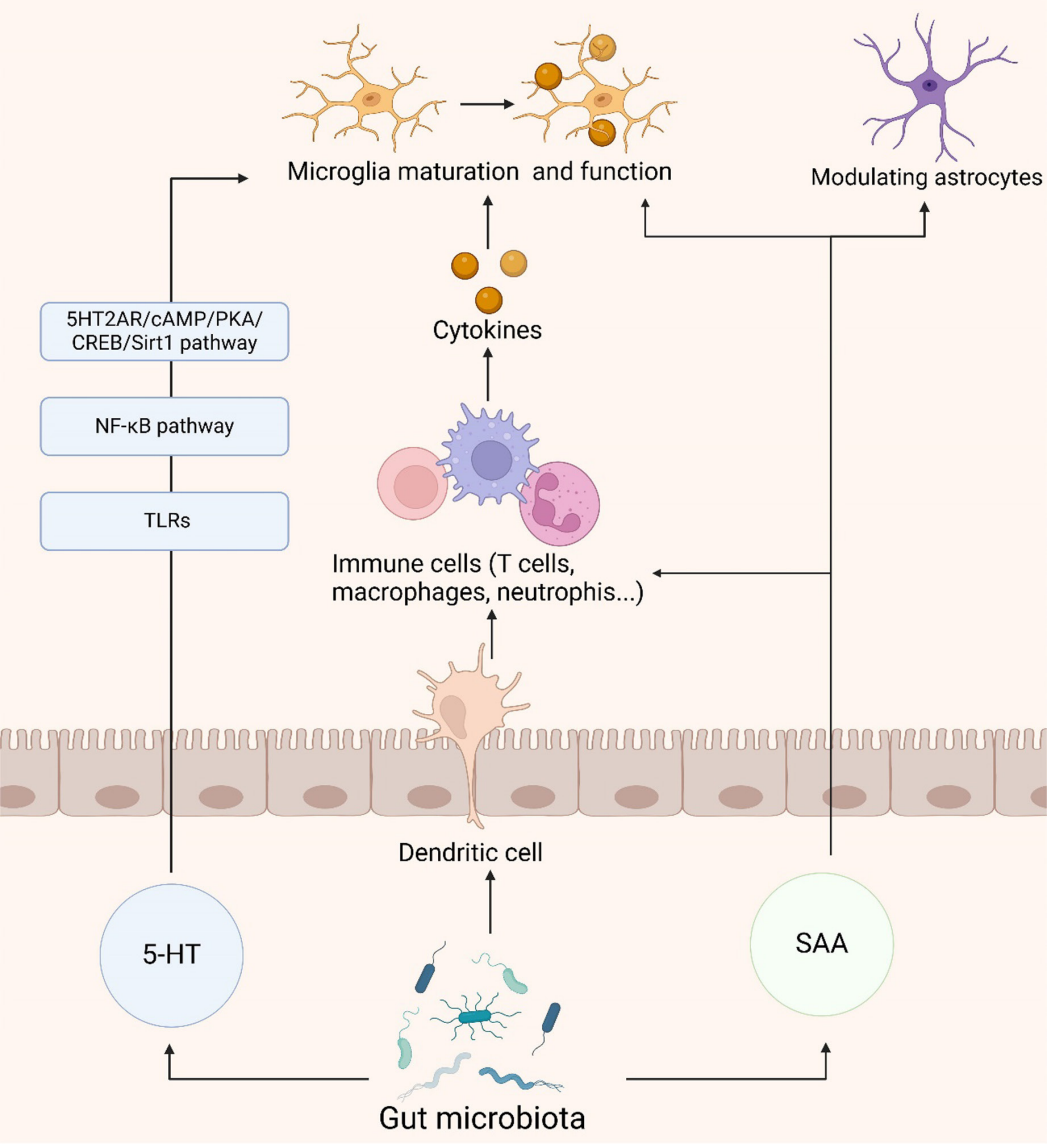
The immune pathway of the gut microbiota in AD. The gut microbiota and its produces (5‐HT and SAA), which affect the immune system and glias cells via immune pathway. This figure was created with BioRender.com. 5‐HT, 5‐Hydroxytryptamine (Serotonin); 5HT2AR, 5‐Hydroxytryptamine 2A Receptor; cAMP, Cyclic Adenosine Monophosphate; CREB, CAMP Response Element‐Binding Protein; NF‐κB, Nuclear Factor kappa‐light‐chain‐enhancer of activated B cells; PKA, Protein Kinase A; SAA, Serum Amyloid A; Sirt1, Sirtuin 1; TLRs, Toll‐Like Receptors.

In this immune pathway, the intestinal microbiota has a role beyond immune system modulation. Its regulation of 5‐HT, mediation of inflammatory responses through SAA and interactions with neurons provide insights into the complex pathogenesis of neuroinflammation (Figure 4). These findings not only enhance our understanding of neuroimmune interactions but also suggest new avenues for potential therapeutic strategies to mitigate neuroinflammatory processes.

### 3.3 The Vagus Nerve Pathway

**FIGURE 5:**
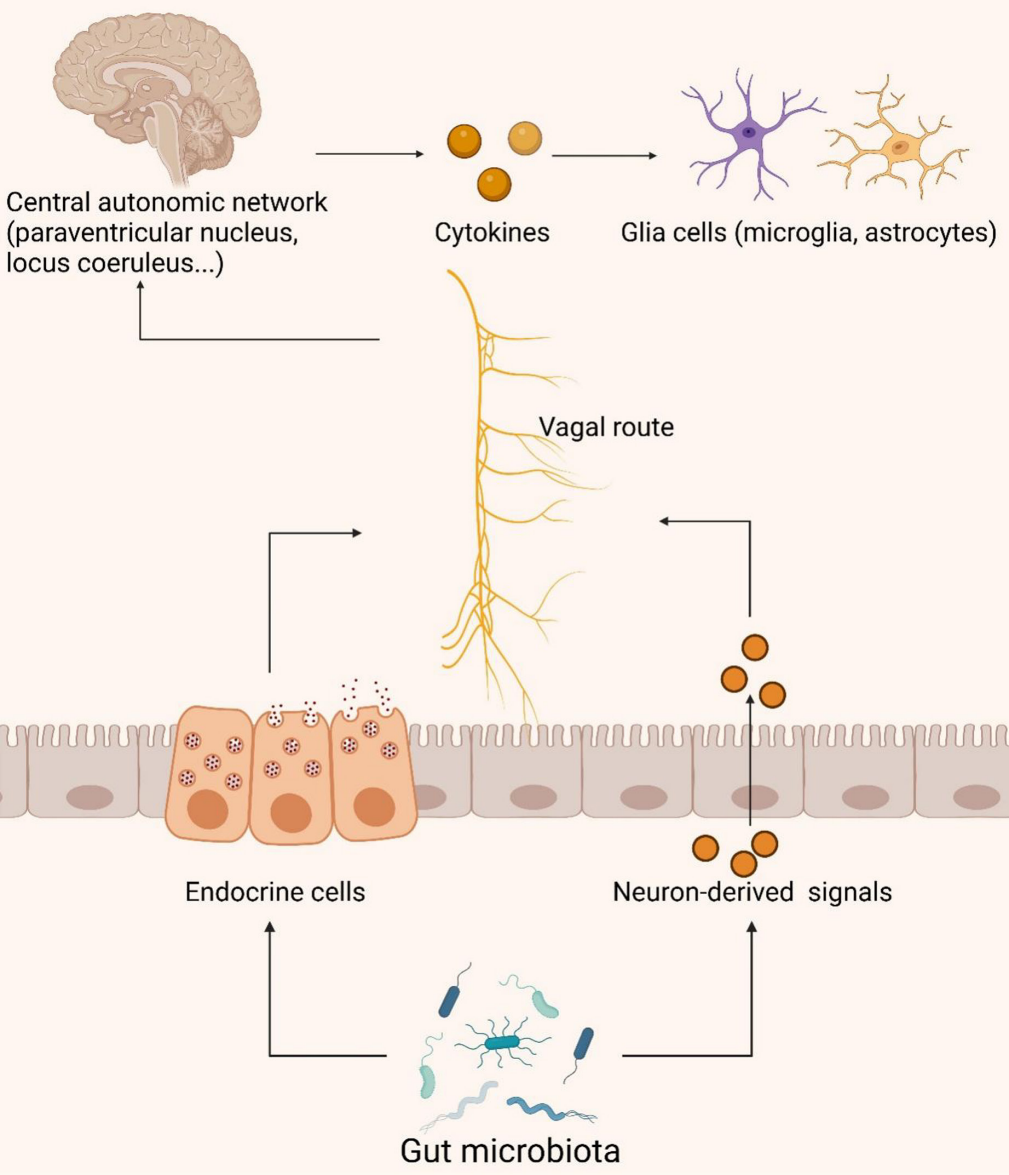
The vagus nerve pathway of the gut microbiota in AD. Gut endocrine cells relay information to the central autonomic network of the brain through interactions with afferent fibers of the vagus nerve. Activation of the vagus nerve modulates the levels of inflammatory signaling molecules, which govern the function of microglia and astrocytes. This figure was created with BioRender.com.

The vagus nerve (VN), a pivotal component of the autonomic nervous system, comprises 80% afferent fibers and 20% efferent fibers. It intricately traverses the gastrointestinal tract, functioning as a critical neural pathway [136]. By facilitating bidirectional information transmission with visceral organs via motor and sensory fibers, the VN regulates organ function and maintains internal organismal homeostasis [137]. This nerve serves as a pivotal link between the intestinal microbiota and the brain, playing a crucial role in the neural‐immune and gut–brain axes (Figure 5) [137].

Intestinal endocrine cells interact directly with VN afferent fibers, transmitting information to the central autonomic network for analysis and integration (including the paraventricular nucleus, locus coeruleus, hypothalamus, and limbic system encompassing the thalamus, amygdala, and hippocampus) [138]. Research indicates that chronic VN stimulation in rats with AD can enhance their memory, likely through modulation of glutamate receptors [139]. VN stimulation activates the locus coeruleus, triggering catecholamine release in the hippocampus and neocortex, enhancing synaptic plasticity, and reducing levels of inflammatory signaling factors (such as TNF‐α, IL‐1β, and IL‐6) [140, 141, 142]. These pro‐inflammatory molecules may access the CNS via the bloodstream or VN afferent fibers, triggering neuroinflammatory responses, activating microglial cells and astrocytes, and leading to neuronal damage and cognitive impairment [143, 144, 145]. Furthermore, VN efferent fibers can synthesize and release acetylcholine (ACh), influencing cholinergic neurons. Activation of ACh released by VN efferent fibers inhibits TNF‐α secretion, demonstrating an anti‐inflammatory effect through binding to α‐7 nicotinic ACh receptors on macrophages [146]. While the VN's role in the gastrointestinal domain is clear, the exact operational pathways and complex processes are still under investigation. Concurrently, increasing research underscores the VN's significant role in elucidating the interplay between gut microbiota and neuroinflammation.

### 3.4 The Gut–Brain Barrier

**FIGURE 6:**
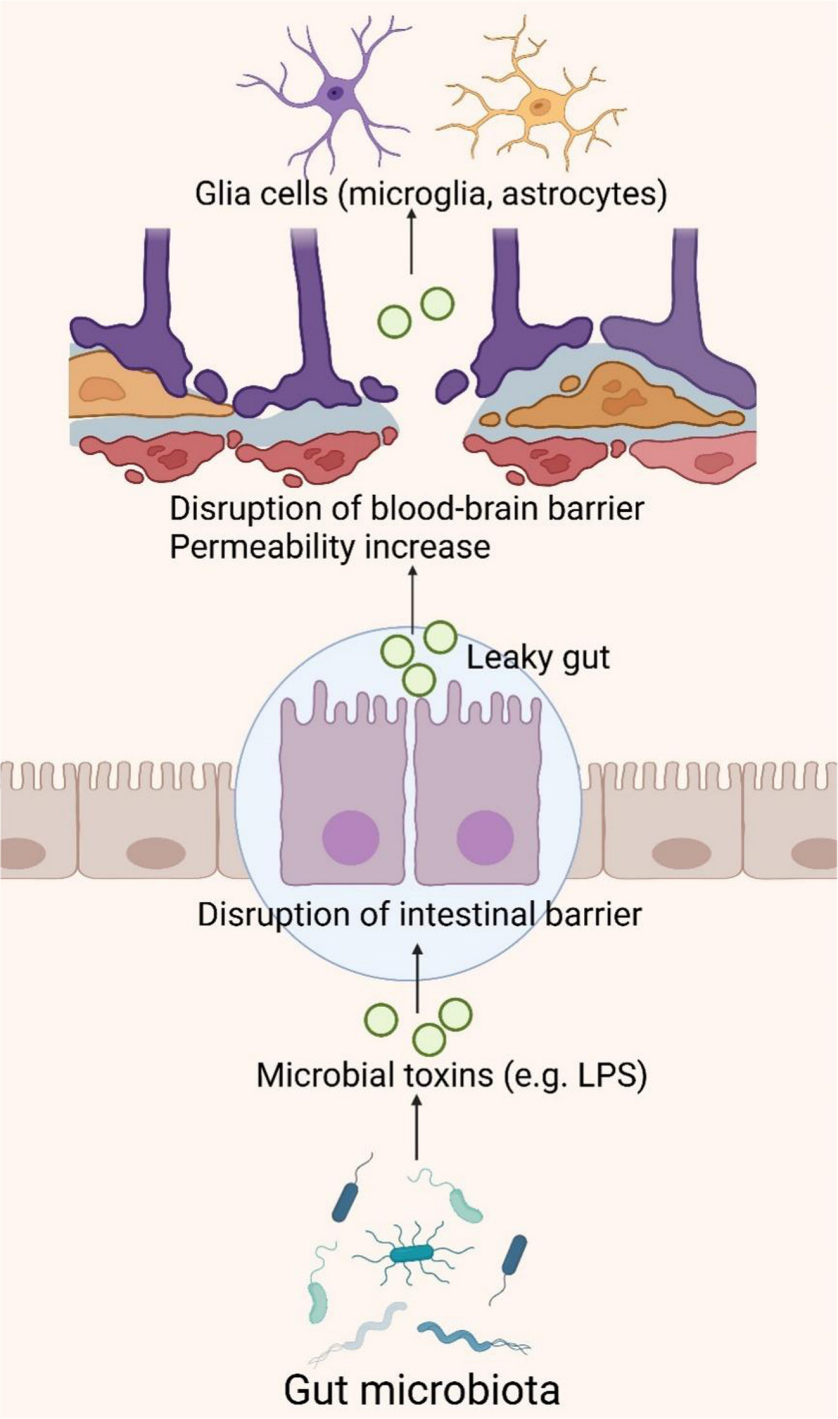
The gut–brain barrier pathway of the gut microbiota in AD. The dysbiosis of gut microbiota results in the production of lipopolysaccharides (LPS) and other toxins, which compromise intestinal barrier integrity, thereby increasing gut permeability. Consequently, these toxins enter the bloodstream and ultimately disrupt the blood–brain barrier, heightening its permeability and allowing more inflammatory mediators and toxins to infiltrate the brain. LPS activates astrocytes and microglia, eliciting the release of various cytokines and chemokines, culminating in neuroinflammation. This figure was created with BioRender.com.

The IB consists of the epithelial layer that lines the intestinal tract, along with associated elements such as the mucous layer, tight junctions, and immune cells, orchestrating the selective passage of intestinal contents to safeguard against pathogens and toxins [147]. The BBB is constituted by specialized brain endothelial cells within microvessels, meticulously regulating the exchange of molecules and nutrients between the bloodstream and brain tissue [148]. Dysbiosis of the intestinal microbiota, characterized by reduced diversity, inflammation, and toxicity, compromises IB integrity, potentially triggering or exacerbating inflammation at the IB and permitting the unchecked translocation of pathogenic microbiota across the BBB (Figure 6) [149]. Persistent systemic inflammation can perturb BBB structure, increasing permeability and precipitating neuroinflammation, neurodegeneration, and age‐related cerebral changes [150, 151].

Specific Gram‐negative bacteria in the gut can produce substantial quantities of amyloids, lipopolysaccharides (LPS), or endotoxins, breaching the IB and BBB to provoke robust pro‐inflammatory and innate immune responses within the CNS, potentially modulating signaling pathways and pro‐inflammatory cytokine production associated with AD [152, 153, 154]. LPS, a prevalent endotoxin, acts as an immunostimulant by being transported to the surface of myeloid cells via LBP, where they bind to membrane‐bound CD14, forming a complex that subsequently activates the TLR4‐MD2 complex. The activation of TLR4 initiates a signaling cascade involving MyD88, IRAK, and TRAF6, ultimately leading to the activation of NF‐κB and MAPK through the NIK and TAK1 pathways. This cascade results in the release of inflammatory cytokines such as IL‐1β, IL‐6, and TNF‐α, thereby instigating an inflammatory response [155]. Research has identified the accumulation of bacterial‐derived LPS within the neuronal parenchyma and around the periphery of neuronal nuclei in the hippocampus and superior temporal gyrus of AD patients [156, 157]. This occurrence is attributed to the synergistic effects of bacterial LPS and amyloid‐like proteins, which may exacerbate intestinal permeability, leading to elevated cytokine levels such as IL‐17A and IL‐22, known to correlate with AD [158, 159]. Moreover, LPS stimulation of the enteric nervous system induces the production of pro‐inflammatory cytokines TNF‐α, IL‐1β, and IL‐6, selectively activating TLR4 on astrocytes and microglia, triggering NF‐κB pathway activation, escalating cytokine production, inducing neuroinflammation, and fostering Aβ deposition, crucial in inflammatory signaling associated with AD [160, 161, 162]. This triggers NF‐κB pathway activation, increasing cytokine production, inducing neuroinflammation, and Aβ deposition, playing a key role in the inflammatory signaling cascade in AD patients [163, 164, 165]. Additionally, during aging, vascular impairments, or degenerative diseases, harmful metabolites originating from the gut microbiota may permeate into the systemic circulation and cerebrovascular system, accumulating at both systemic and cerebral levels [166]. This accumulation can elevate ROS and activate the NF‐κB signaling pathway, thereby upregulating pro‐inflammatory miRNA‐34a. Consequently, this downregulates TREM2 expression, impairing microglial phagocytic function and resulting in Aβ accumulation [158, 159].

## 4 Prospective Therapeutic Strategies for AD

**FIGURE 7:**
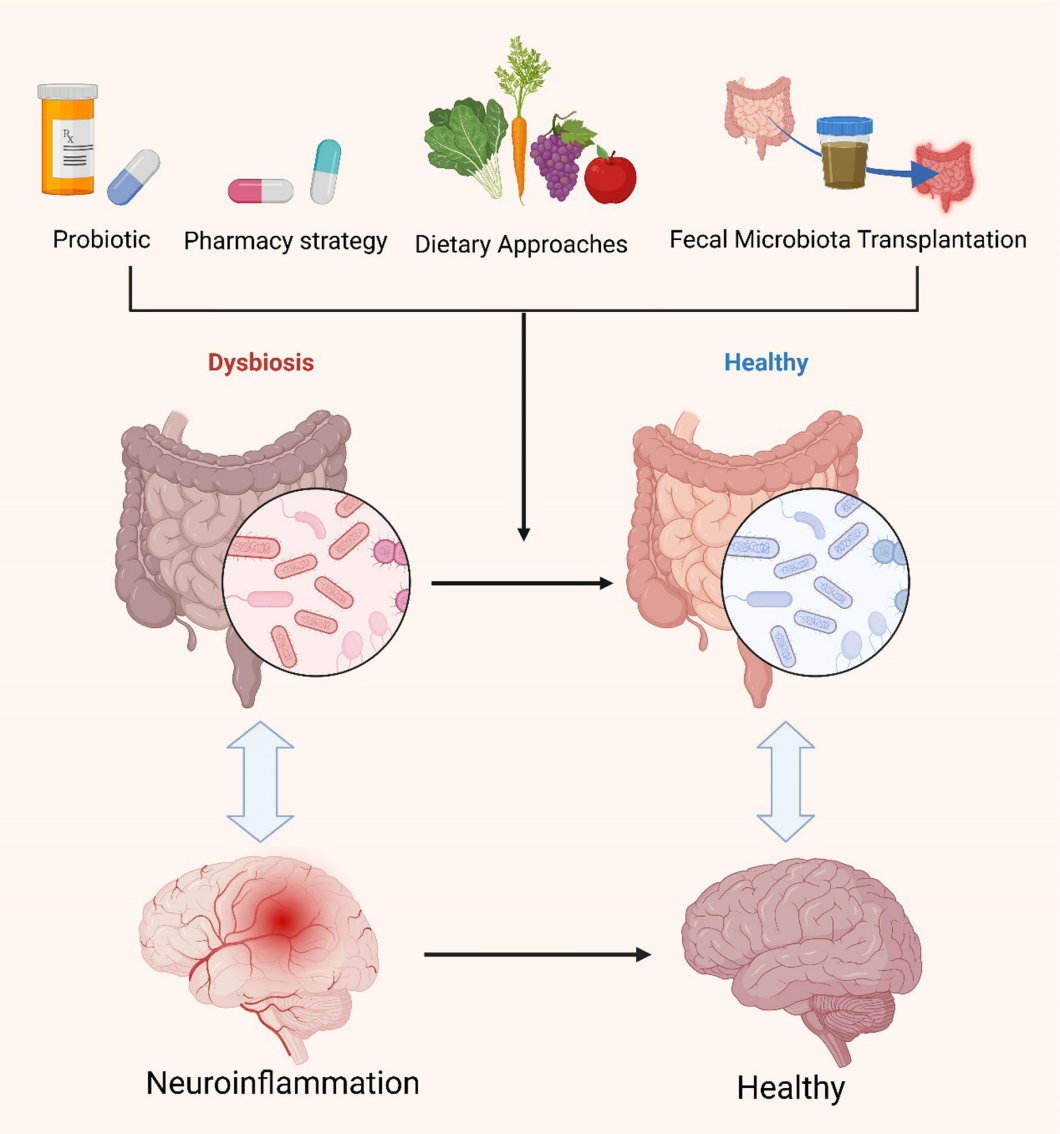
Microbiome‐targeted therapeutic approaches for AD. Emerging evidence from both murine and human studies indicates that interventions such as probiotics, prebiotics, fecal microbiota transplantation from healthy donors to AD patients, microbiome‐modulating pharmacological agents, and the direct targeting of gut microbiota‐regulated neuroinflammatory pathways hold promise as potential disease‐modifying therapies with the capacity to mitigate neurodegeneration. This figure was created with BioRender.com.

The intricate mechanisms by which the gut microbiota influences AD pathogenesis offer promising avenues for future therapeutic interventions. Compared to conventional brain‐targeted treatments, strategies focusing on the gut microbiota possess unique advantages, not only circumventing the challenge of the BBB but also allowing for more precise modulation of host–microbe interactions, thereby achieving faster therapeutic outcomes. These approaches are also regarded as safer, given their lower side effect profile, particularly through interventions such as dietary modifications, supplementation with probiotics and prebiotics, fecal microbiota transplantation, and other microbiota modulators, which can effectively reshape the composition of the gut microbiome, yielding beneficial effects on neurological disorders and alleviating pathological conditions (Figure 7; Table 2) [10, 35, 38, 138, 167, 168, 169, 170, 171, 172, 173, 174, 175, 176, 177, 178]. Moreover, an increasing body of research suggests that certain traditional Chinese herbal monomers, extracts, and compound formulations may exert potential preventive and therapeutic effects in AD by modulating the composition, diversity, and abundance of the gut microbiota. This further underscores the importance of the gut–brain axis in neurodegenerative diseases and highlights that gut microbiota‐targeted therapies represent not only an emerging field in AD treatment but also a novel perspective for enhancing overall health.

**TABLE 2:** Therapeutic approaches targeting gut microbiota in AD.

Strategy	Model	Therapeutic method	Therapeutic outcome	Refs.
FMT	APP/PS1 transgenic mice	Transplantation of fecal microbiota from wild‐type mice to transgenic mice expressing APP, PSEN1, and MAPT	Improvement in pathological Aβ plaques, neurofibrillary tangles, neuroglial reactivity, and cognitive impairment	[170]
	82‐year‐old male AD patient	FMT from the patient's cognitively sharp wife	Rapid improvement in AD symptoms (significant increase in MMSE score)	[35]
	90‐year‐old female AD patient	FMT from a 27‐year‐old male	Significant improvement in MMSE, MoCA, and CDR scores	[171]
Dietary approaches	Sprague–Dawley rats	Dietary fiber	Interference with the formation of soluble neurotoxic Aβ aggregates	[174]
	APOE4 transgenic mice	Dietary inulin	Elevation of gastrointestinal microbiota metabolites (SCFAs, tryptophan‐derived metabolites, bile acids); reduction in hippocampal inflammation gene expression	[173]
Probiotic	Elderly individuals (≥ 65 years old)	Probiotic supplementation(BGN4 and BORI)	Heightened serum levels of BDNF	[175]
	3xTg‐AD mice	Lactobacillus lactis carrying plasmid encoding human p62 protein	Improved memory, regulated ubiquitin‐proteasome system and autophagy; reduced amyloid burden, oxidative stress, and neuroinflammation	[176]
	AppNL‐G‐F mice	Lactic acid‐producing bacteria	Reduced gut inflammation and permeability; minimal impact on brain plaque deposition or cognitive improvement	[177]
	Mice injected intracerebroventricularly with Aβ25‐35	Bifidobacterium breve strain A1	Decreased expression of inflammation and immune response genes in the hippocampus	[141]
	3xTg‐AD Mice	Probiotic formulation (SLAB51)	Reduced Aβ aggregation and partially restored compromised neuronal proteins	[140]
	*C. elegans*	Lactobacillus rhamnosus HA‐114	Demonstrated neuroprotective properties	[178]
Pharmacy strategy	5XFAD transgenic mice	GV‐971 (sodium oligo‐mannurarate)	Attenuated intestinal dysbiosis and regulated neuroinflammation	[169]
	APP/PS1 transgenic mice	Long‐term administration of broad‐spectrum antibiotics	Reduced amyloid plaque deposition, increased soluble Aβ levels; mitigated neuroinflammatory responses by reducing plaque‐associated gliosis and altering microglial morphology	[172]

Abbreviations: AD, Alzheimer's disease; APP, amyloid precursor protein; Aβ, amyloid‐β; BDNF, brain‐derived neurotrophic factor; CDR, clinical dementia rating; FMT, fecal microbiota transplantation; MAPT, microtubule‐associated protein Tau; MMSE, mini‐mental state examination; MoCA, Montreal cognitive assessment; PSEN1, Presenilin‐1; SCFAs, short‐chain fatty acids.

## 5 Conclusions and Discussion

AD is witnessing an alarming global surge in prevalence, with the foremost challenge to treatment lying in the incomplete elucidation of its pathogenesis. Current FDA‐approved therapies offer only marginal benefits, emphasizing the critical need to explore innovative therapeutic strategies and targets. Inflammatory signals are central to AD pathogenesis, mediated by the bidirectional communication of the gut–brain axis. This study delves into the intricate ways in which gut microbiota modulates AD progression through diverse mechanisms such as metabolite production, immune regulation, preservation of intestinal and BBB integrity, and neurotransmitter synthesis.

The vital role of a healthy gut microbiota in maintaining immune homeostasis includes: (i) fermenting dietary fibers to produce SCFAs, which regulate immune responses by binding to receptors on both intestinal and peripheral immune cells, thereby promoting the secretion of anti‐inflammatory cytokines and inhibiting pro‐inflammatory factors. This immunomodulatory effect reduces systemic inflammation, which in turn alleviates neuroinflammation; (ii) It helps preserve the integrity of the intestinal epithelial barrier, preventing harmful substances such as pathogens and toxins from entering the bloodstream; (iii) It fosters the generation of Tregs, reducing the release of pro‐inflammatory cytokines; and (iv) Through the BBB, it modulates microglial activation, keeping them in an anti‐inflammatory and tissue repair mode, thereby diminishing neuroinflammation. Conversely, dysbiosis promotes chronic, low‐grade inflammation, exacerbating neuroinflammatory pathways linked to attention deficit disorder [37, 157, 169, 170]. These mechanisms underscore the pivotal role of the gut microbiota in regulating both local and systemic immune responses, thus influencing the trajectory of neuroinflammation and attention deficit disorder. A well‐balanced gut microbiota supports anti‐inflammatory processes and preserves the integrity of the gut barrier, which is crucial in preventing systemic inflammation from impacting the brain [155, 179, 180]. These mechanisms highlight the critical role of gut microbiota in modulating both local and systemic immune responses, consequently shaping neuroinflammation and the trajectory of AD progression. Dysbiosis has been linked to the promotion of neuroinflammation, while a balanced microbiome appears to offer neuronal protection and mitigate the advancement of AD. Although this paper predominantly focuses on bacteria, it is imperative to recognize that the gut microbiota encompasses viruses, phages, fungi, and other microorganisms, whose roles in AD pathogenesis merit further investigation. Mechanistic insights into how gut microbiota metabolites impact human health, alongside translational research and multifactorial studies—including age, diet, ethnicity, environment, and physical activity—remain crucial areas for future exploration [181].

In conclusion, this study underscores the fundamental influence of gut microbiota on AD pathogenesis, advocating for a deeper investigation into its diversity and impact as a driver for more efficacious therapeutic interventions. In particular, further clarification of the interplay between gut microbiota and immune homeostasis is necessary, as chronic inflammation stemming from dysbiosis plays a central role in exacerbating neuroinflammation and neuronal damage in AD. Future studies should aim to establish causal links between gut microbiota and AD, paving the way for novel microbiome‐targeted treatments that may alter the disease's course.

## 6 Current Challenges and Future Perspectives

The significance of the gut microbiota to human health was recognized by scientists over a century ago, but research was hindered by limited methodologies, particularly the inability to culture anaerobic bacteria within the gut microbiome. The advent of modern technologies, such as anaerobic culturing, DNA fingerprinting, next‐generation sequencing, and real‐time quantitative PCR, has significantly advanced the study of the gut microbiota [182, 183]. At this stage, defining a healthy microbiota is exceedingly challenging. The abundance and diversity of gut microbiota exhibit considerable individual variability (e.g., gender, race, genetic background, environmental factors, dietary habits, etc.), necessitating further research employing metagenomic analysis and integrating multiple omics approaches such as proteomics, genomics, and metabolomics, rather than solely relying on 16S rRNA gene sequencing to elucidate the regularity of gut microbiota structure and strain levels in AD patients. For high‐risk populations, such as individuals with a family history of AD and the elderly, more research on the effects of microbiota‐based interventions for AD, potential interactions with other therapies, appropriate sample sizes, and longer follow‐up studies should be considered. Currently, methods for modulating the gut microbiota mainly include probiotics, prebiotics, fecal microbiota transplantation, antibiotics, etc. However, the efficacy and safety of these methods require further clinical trials and long‐term observations for validation. Additionally, when administering drugs, it is crucial to consider the impact of the medication on other microbiota interventions. It is worth noting that although therapeutic approaches targeting the gut microbiota have certain advantages, strategies for gut microbiota modulation in AD are still in the research stage. Despite some preliminary research suggesting the potential benefits of gut microbiota modulation for AD, the clinical translation of microbiome‐based therapies remains challenging, thus requiring continuous research efforts to unravel the complexity of the microbiota–gut–brain axis and fully exploit its potential. With the increasing maturity of technology and methodological innovations, further exploration of the causal relationship between the gut microbiota and AD, elucidation of the molecular mechanisms of microbiota–gut–brain axis neuroinflammation regulation, discovery of more gut microbiota‐related AD biomarkers, and development of more effective and personalized gut microbiota modulation therapies are warranted. The plasticity of the human gut microbiome provides an exciting opportunity for the development of personalized microbiota‐based therapies for AD.

## Conflicts of Interest

The authors declare no conflicts of interest.

## Data Availability

The authors have nothing to report.

## References

[1] J. M. Long and D. M. Holtzman , “Alzheimer Disease: An Update on Pathobiology and Treatment Strategies,” Cell 179 (2019): 312–339. 31564456 10.1016/j.cell.2019.09.001 PMC6778042

[2] Alzheimer's Association Report , “2023 Alzheimer's Disease Facts and Figures,” Alzheimers Dement 19 (2023): 1598–1695. 36918389 10.1002/alz.13016

[3] T. Guo , D. Zhang , Y. Zeng , T. Y. Huang , H. Xu , and Y. Zhao , “Molecular and Cellular Mechanisms Underlying the Pathogenesis of Alzheimer's Disease,” Molecular Neurodegeneration 15 (2020): 40. 32677986 10.1186/s13024-020-00391-7 PMC7364557

[4] C. S. Subhramanyam , C. Wang , Q. Hu , and S. T. Dheen , “Microglia‐Mediated Neuroinflammation in Neurodegenerative Diseases,” Seminars in Cell & Developmental Biology 94 (2019): 112–120. 31077796 10.1016/j.semcdb.2019.05.004

[5] M. T. Heneka , M. J. Carson , J. El Khoury , et al., “Neuroinflammation in Alzheimer's Disease,” Lancet Neurology 14 (2015): 388–405. 25792098 10.1016/S1474-4422(15)70016-5 PMC5909703

[6] C. G. Hart and S. Karimi‐Abdolrezaee , “Recent Insights on Astrocyte Mechanisms in CNS Homeostasis, Pathology, and Repair,” Journal of Neuroscience Research 99 (2021): 2427–2462. 34259342 10.1002/jnr.24922

[7] X. Zhang , H. Zhong , Y. Li , et al., “Sex‐ and Age‐Related Trajectories of the Adult Human Gut Microbiota Shared Across Populations of Different Ethnicities,” Nature Aging 1 (2021): 87–100. 37118004 10.1038/s43587-020-00014-2

[8] C. Jiang , G. Li , P. Huang , Z. Liu , and B. Zhao , “The Gut Microbiota and Alzheimer's Disease,” Journal of Alzheimer's Disease 58 (2017): 1–15. 10.3233/JAD-161141 28372330

[9] S. Liu , J. Gao , M. Zhu , K. Liu , and H. L. Zhang , “Gut Microbiota and Dysbiosis in Alzheimer's Disease: Implications for Pathogenesis and Treatment,” Molecular Neurobiology 57 (2020): 5026–5043. 32829453 10.1007/s12035-020-02073-3 PMC7541367

[10] Y. Fan and O. Pedersen , “Gut Microbiota in Human Metabolic Health and Disease,” Nature Reviews. Microbiology 19 (2021): 55–71. 32887946 10.1038/s41579-020-0433-9

[11] A. A. Vandenbark , H. Offner , S. Matejuk , and A. Matejuk , “Microglia and Astrocyte Involvement in Neurodegeneration and Brain Cancer,” Journal of Neuroinflammation 18 (2021): 298. 34949203 10.1186/s12974-021-02355-0 PMC8697466

[12] B. Dalile , L. Van Oudenhove , B. Vervliet , and K. Verbeke , “The Role of Short‐Chain Fatty Acids in Microbiota‐Gut‐Brain Communication,” Nature Reviews. Gastroenterology & Hepatology 16 (2019): 461–478. 31123355 10.1038/s41575-019-0157-3

[13] L. H. Morais , H. L. Schreiber , and S. K. Mazmanian , “The Gut Microbiota–Brain Axis in Behaviour and Brain Disorders,” Nature Reviews Microbiology 19 (2021): 241–255. 33093662 10.1038/s41579-020-00460-0

[14] E. Elinav , T. Strowig , A. L. Kau , et al., “NLRP6 Inflammasome Regulates Colonic Microbial Ecology and Risk for Colitis,” Cell 145 (2011): 745–757. 21565393 10.1016/j.cell.2011.04.022 PMC3140910

[15] D. Li , S. Yu , Y. Long , et al., “Tryptophan Metabolism: Mechanism‐Oriented Therapy for Neurological and Psychiatric Disorders,” Frontiers in Immunology 13 (2022): 985378. 36159806 10.3389/fimmu.2022.985378 PMC9496178

[16] D. J. Selkoe and J. Hardy , “The Amyloid Hypothesis of Alzheimer's Disease at 25 Years,” EMBO Molecular Medicine 8 (2016): 595–608. 27025652 10.15252/emmm.201606210 PMC4888851

[17] W. Y. Wang , M. S. Tan , J. T. Yu , and L. Tan , “Role of Pro‐Inflammatory Cytokines Released From Microglia in Alzheimer's Disease,” Annals of Translational Medicine's 3 (2015): 136. 10.3978/j.issn.2305-5839.2015.03.49 PMC4486922 26207229

[18] Q. Wang , H. Yao , W. Liu , et al., “Microglia Polarization in Alzheimer's Disease: Mechanisms and a Potential Therapeutic Target,” Frontiers in Aging Neuroscience 13 (2021): 772717. 34819850 10.3389/fnagi.2021.772717 PMC8606412

[19] E. Zenaro , G. Piacentino , and G. Constantin , “The Blood‐Brain Barrier in Alzheimer's Disease,” Neurobiology of Disease 107 (2017): 41–56. 27425887 10.1016/j.nbd.2016.07.007 PMC5600438

[20] Z. Cai , P. F. Qiao , C. Q. Wan , M. Cai , N. K. Zhou , and Q. Li , “Role of Blood‐Brain Barrier in Alzheimer's Disease,” Journal of Alzheimer's Disease 63 (2018): 1223–1234. 10.3233/JAD-180098 29782323

[21] A. Picca , J. Faitg , J. Auwerx , L. Ferrucci , and D. D'Amico , “Mitophagy in Human Health, Ageing and Disease,” Nature Metabolism 5 (2023): 2047–2061. 10.1038/s42255-023-00930-8 PMC12159423 38036770

[22] J. Liang , B. Liu , X. Dong , et al., “Decoding the Role of Gut Microbiota in Alzheimer's Pathogenesis and Envisioning Future Therapeutic Avenues,” Frontiers in Neuroscience 17 (2023): 1242254. 37790586 10.3389/fnins.2023.1242254 PMC10544353

[23] J. S. Johnson , D. J. Spakowicz , B. Y. Hong , et al., “Evaluation of 16S rRNA Gene Sequencing for Species and Strain‐Level Microbiome Analysis,” Nature Communications 10 (2019): 5029. 10.1038/s41467-019-13036-1 PMC6834636 31695033

[24] C. Wang , J. Zhao , H. Zhang , Y. K. Lee , Q. Zhai , and W. Chen , “Roles of Intestinal Bacteroides in Human Health and Diseases,” Critical Reviews in Food Science and Nutrition 61 (2021): 3518–3536. 32757948 10.1080/10408398.2020.1802695

[25] C. Y. L. Chong , F. H. Bloomfield , and J. M. O'Sullivan , “Factors Affecting Gastrointestinal Microbiome Development in Neonates,” Nutrients 10 (2018): 274. 29495552 10.3390/nu10030274 PMC5872692

[26] M. Carabotti , A. Scirocco , M. A. Maselli , and C. Severi , “The Gut‐Brain Axis: Interactions Between Enteric Microbiota, Central and Enteric Nervous Systems,” Annals of Gastroenterology 28 (2015): 203–209. 25830558 PMC4367209

[27] D. C. Emery , D. K. Shoemark , T. E. Batstone , et al., “16S rRNA Next Generation Sequencing Analysis Shows Bacteria in Alzheimer's Post‐Mortem Brain,” Frontiers in Aging Neuroscience 9 (2017): 195. 28676754 10.3389/fnagi.2017.00195 PMC5476743

[28] N. M. Vogt , R. L. Kerby , K. A. Dill‐McFarland , et al., “Gut Microbiome Alterations in Alzheimer's Disease,” Scientific Reports 7 (2017): 13537. 29051531 10.1038/s41598-017-13601-y PMC5648830

[29] A. Cattaneo , N. Cattane , S. Galluzzi , et al., “Association of Brain Amyloidosis With Pro‐Inflammatory Gut Bacterial Taxa and Peripheral Inflammation Markers in Cognitively Impaired Elderly,” Neurobiology of Aging 49 (2017): 60–68. 27776263 10.1016/j.neurobiolaging.2016.08.019

[30] L. Whiley , K. E. Chappell , E. D'Hondt , et al., “Metabolic Phenotyping Reveals a Reduction in the Bioavailability of Serotonin and Kynurenine Pathway Metabolites in Both the Urine and Serum of Individuals Living With Alzheimer's Disease,” Alzheimer's Research & Therapy 13 (2021): 20. 10.1186/s13195-020-00741-z PMC7797094 33422142

[31] X. Zhan , B. Stamova , L. W. Jin , C. DeCarli , B. Phinney , and F. R. Sharp , “Gram‐Negative Bacterial Molecules Associate With Alzheimer Disease Pathology,” Neurology 87 (2016): 2324–2332. 27784770 10.1212/WNL.0000000000003391 PMC5135029

[32] B. J. H. Verhaar , H. M. A. Hendriksen , F. A. de Leeuw , et al., “Gut Microbiota Composition Is Related to AD Pathology,” Frontiers in Immunology 12 (2021): 794519. 35173707 10.3389/fimmu.2021.794519 PMC8843078

[33] N. Salazar , S. Arboleya , T. Fernández‐Navarro , C. G. de Los Reyes‐Gavilán , S. Gonzalez , and M. Gueimonde , “Age‐Associated Changes in Gut Microbiota and Dietary Components Related With the Immune System in Adulthood and Old Age: A Cross‐Sectional Study,” Nutrients 11 (2019): 1765. 31370376 10.3390/nu11081765 PMC6722604

[34] E. Biagi , L. Nylund , M. Candela , et al., “Through Ageing, and Beyond: Gut Microbiota and Inflammatory Status in Seniors and Centenarians,” PLoS One 5 (2010): e10667. 20498852 10.1371/journal.pone.0010667 PMC2871786

[35] S. Hazan , “Rapid Improvement in Alzheimer's Disease Symptoms Following Fecal Microbiota Transplantation: A Case Report,” Journal of International Medical Research 48 (2020): 300060520925930. 32600151 10.1177/0300060520925930 PMC7328362

[36] K. C. Fan , C. C. Lin , Y. C. Liu , et al., “Altered Gut Microbiota in Older Adults With Mild Cognitive Impairment: A Case‐Control Study,” Frontiers in Aging Neuroscience 15 (2023): 1162057. 37346147 10.3389/fnagi.2023.1162057 PMC10281289

[37] D. Erny , A. L. Hrabě de Angelis , D. Jaitin , et al., “Host Microbiota Constantly Control Maturation and Function of Microglia in the CNS,” Nature Neuroscience 18 (2015): 965–977. 26030851 10.1038/nn.4030 PMC5528863

[38] J. Sun , J. Xu , Y. Ling , et al., “Fecal Microbiota Transplantation Alleviated Alzheimer's Disease‐Like Pathogenesis in APP/PS1 Transgenic Mice,” Translational Psychiatry 9 (2019): 189. 31383855 10.1038/s41398-019-0525-3 PMC6683152

[39] L. Zhang , Y. Wang , X. Xiayu , et al., “Altered Gut Microbiota in a Mouse Model of Alzheimer's Disease,” Journal of Alzheimer's Disease 60 (2017): 1241–1257. 10.3233/JAD-170020 29036812

[40] C. Brandscheid , F. Schuck , S. Reinhardt , et al., “Altered Gut Microbiome Composition and Tryptic Activity of the 5xFAD Alzheimer's Mouse Model,” Journal of Alzheimer's Disease 56 (2017): 775–788. 10.3233/JAD-160926 28035935

[41] C. Chen , Y. Zhou , H. Wang , et al., “Gut Inflammation Triggers C/EBPβ/δ‐Secretase‐Dependent Gut‐To‐Brain Propagation of Aβ and Tau Fibrils in Alzheimer's Disease,” EMBO Journal 40, no. 17 (2021): e106320. 34260075 10.15252/embj.2020106320 PMC8408610

[42] C. Chen , J. Liao , Y. Xia , et al., “Gut Microbiota Regulate Alzheimer's Disease Pathologies and Cognitive Disorders via PUFA‐Associated Neuroinflammation,” Gut 71 (2022): 2233–2252. 35017199 10.1136/gutjnl-2021-326269 PMC10720732

[43] B. L. Sun , W. W. Li , J. Wang , et al., “Gut Microbiota Alteration and Its Time Course in a Tauopathy Mouse Model,” Journal of Alzheimer's Disease 70 (2019): 399–412. 10.3233/JAD-181220 31177213

[44] S. Elangovan and T. J. Borody , “Holsinger RMD: Fecal Microbiota Transplantation Reduces Pathology and Improves Cognition in a Mouse Model of Alzheimer's Disease,” Cells 12 (2022): 12. 36611911 10.3390/cells12010119 PMC9818266

[45] H. Shen , Q. Guan , X. Zhang , et al., “New Mechanism of Neuroinflammation in Alzheimer's Disease: The Activation of NLRP3 Inflammasome Mediated by Gut Microbiota,” Progress in Neuro‐Psychopharmacology & Biological Psychiatry 100 (2020): 109884. 32032696 10.1016/j.pnpbp.2020.109884

[46] Y. Xia , Y. Xiao , Z. H. Wang , et al., “Bacteroides Fragilis in the Gut Microbiomes of Alzheimer's Disease Activates Microglia and Triggers Pathogenesis in Neuronal C/EBPβ Transgenic Mice,” Nature Communications 14 (2023): 5471. 10.1038/s41467-023-41283-w PMC10482867 37673907

[47] B. Li , Y. He , J. Ma , et al., “Mild Cognitive Impairment Has Similar Alterations as Alzheimer's Disease in Gut Microbiota,” Alzheimers Dement 15 (2019): 1357–1366. 31434623 10.1016/j.jalz.2019.07.002

[48] D. Bairamian , S. Sha , N. Rolhion , et al., “Microbiota in Neuroinflammation and Synaptic Dysfunction: A Focus on Alzheimer's Disease,” Molecular Neurodegeneration 17 (2022): 19. 35248147 10.1186/s13024-022-00522-2 PMC8898063

[49] L. Wu , Y. Han , Z. Zheng , et al., “Altered Gut Microbial Metabolites in Amnestic Mild Cognitive Impairment and Alzheimer's Disease: Signals in Host‐Microbe Interplay,” Nutrients 13 (2021): 228. 33466861 10.3390/nu13010228 PMC7829997

[50] C. Gao , B. Li , Y. He , et al., “Early Changes of Fecal Short‐Chain Fatty Acid Levels in Patients With Mild Cognitive Impairments,” CNS Neuroscience & Therapeutics 29 (2023): 3657–3666. 37144597 10.1111/cns.14252 PMC10580335

[51] N. M. Vogt , K. A. Romano , B. F. Darst , et al., “The Gut Microbiota‐Derived Metabolite Trimethylamine N‐Oxide Is Elevated in Alzheimer's Disease,” Alzheimer's Research & Therapy 10 (2018): 124. 10.1186/s13195-018-0451-2 PMC6303862 30579367

[52] S. Hickman , S. Izzy , P. Sen , L. Morsett , and J. El Khoury , “Microglia in neurodegeneration,” Nature Neuroscience 21 (2018): 1359–1369. 30258234 10.1038/s41593-018-0242-x PMC6817969

[53] J. W. Kinney , S. M. Bemiller , A. S. Murtishaw , A. M. Leisgang , A. M. Salazar , and B. T. Lamb , “Inflammation as a Central Mechanism in Alzheimer's Disease,” Alzheimers Dement (N Y) 4 (2018): 575–590. 30406177 10.1016/j.trci.2018.06.014 PMC6214864

[54] M. Prinz , S. Jung , and J. Priller , “Microglia Biology: One Century of Evolving Concepts,” Cell 179 (2019): 292–311. 31585077 10.1016/j.cell.2019.08.053

[55] H. Zeng , J. Huang , H. Zhou , et al., “Integrative In Situ Mapping of Single‐Cell Transcriptional States and Tissue Histopathology in a Mouse Model of Alzheimer's Disease,” Nature Neuroscience 26 (2023): 430–446. 36732642 10.1038/s41593-022-01251-x PMC11332722

[56] D. P. Wightman , I. E. Jansen , J. E. Savage , et al., “A Genome‐Wide Association Study With 1,126,563 Individuals Identifies New Risk Loci for Alzheimer's Disease,” Nature Genetics 53 (2021): 1276–1282. 34493870 10.1038/s41588-021-00921-z PMC10243600

[57] F. Leng and P. Edison , “Neuroinflammation and Microglial Activation in Alzheimer Disease: Where Do We Go From Here?,” Nature Reviews. Neurology 17 (2021): 157–172. 33318676 10.1038/s41582-020-00435-y

[58] C. S. McAlpine , J. Park , A. Griciuc , et al., “Astrocytic Interleukin‐3 Programs Microglia and Limits Alzheimer's Disease,” Nature 595 (2021): 701–706. 34262178 10.1038/s41586-021-03734-6 PMC8934148

[59] S. Thakur , R. Dhapola , P. Sarma , B. Medhi , and D. H. Reddy , “Neuroinflammation in Alzheimer's Disease: Current Progress in Molecular Signaling and Therapeutics,” Inflammation 46 (2023): 1–17. 35986874 10.1007/s10753-022-01721-1

[60] Y. Chen and Y. Yu , “Tau and Neuroinflammation in Alzheimer's Disease: Interplay Mechanisms and Clinical Translation,” Journal of Neuroinflammation 20 (2023): 165. 37452321 10.1186/s12974-023-02853-3 PMC10349496

[61] S. Moonen , M. J. Koper , E. Van Schoor , et al., “Pyroptosis in Alzheimer's Disease: Cell Type‐Specific Activation in Microglia, Astrocytes and Neurons,” Acta Neuropathologica 145 (2023): 175–195. 36481964 10.1007/s00401-022-02528-y

[62] J. M. Schott , “Infection, Inflammation and Alzheimer's Disease,” European Journal of Neurology: The Official Journal of the European Federation of Neurological Societies 22, no. 12 (2015): 1504.

[63] Z. Xinhua , S. Boryana , and F. R. Sharp , “Lipopolysaccharide Associates With Amyloid Plaques, Neurons and Oligodendrocytes in Alzheimer's Disease Brain: A Review.Frontiers in Aging,” Neuroscience 10 (2018): 42. 10.3389/fnagi.2018.00042 PMC5827158 29520228

[64] G. C. Brown , “The Endotoxin Hypothesis of Neurodegeneration,” Journal of Neuroinflammation 16, no. 1 (2019): 180. 31519175 10.1186/s12974-019-1564-7 PMC6744684

[65] G. C. Brown and M. T. Heneka , “The Endotoxin Hypothesis of Alzheimer's Disease,” Molecular Neurodegeneration 19, no. 1 (2024): 30. 38561809 10.1186/s13024-024-00722-y PMC10983749

[66] K. Kowalski and A. Mulak , “Brain‐Gut‐Microbiota Axis in Alzheimer's Disease,” Journal of Neurogastroenterology and Motility 25 (2019): 48–60. 30646475 10.5056/jnm18087 PMC6326209

[67] A. Koh , F. De Vadder , P. Kovatcheva‐Datchary , and F. Bäckhed , “From Dietary Fiber to Host Physiology: Short‐Chain Fatty Acids as Key Bacterial Metabolites,” Cell 165 (2016): 1332–1345. 27259147 10.1016/j.cell.2016.05.041

[68] M. Marizzoni , A. Cattaneo , P. Mirabelli , et al., “Short‐Chain Fatty Acids and Lipopolysaccharide as Mediators Between Gut Dysbiosis and Amyloid Pathology in Alzheimer's Disease,” Journal of Alzheimer's Disease 78 (2020): 683–697. 10.3233/JAD-200306 33074224

[69] L. Hoyles , T. Snelling , U. K. Umlai , et al., “Microbiome‐Host Systems Interactions: Protective Effects of Propionate Upon the Blood‐Brain Barrier,” Microbiome 6 (2018): 55. 29562936 10.1186/s40168-018-0439-y PMC5863458

[70] J. Wen , Y. Ding , L. Wang , and Y. Xiao , “Gut Microbiome Improves Postoperative Cognitive Function by Decreasing Permeability of the Blood‐Brain Barrier in Aged Mice,” Brain Research Bulletin 164 (2020): 249–256. 32896587 10.1016/j.brainresbull.2020.08.017

[71] J. Liu , Y. Jin , Y. Ye , et al., “The Neuroprotective Effect of Short Chain Fatty Acids Against Sepsis‐Associated Encephalopathy in Mice,” Frontiers in Immunology 12 (2021): 626894. 33584734 10.3389/fimmu.2021.626894 PMC7876449

[72] C. Benakis , C. Martin‐Gallausiaux , J. P. Trezzi , P. Melton , A. Liesz , and P. Wilmes , “The Microbiome‐Gut‐Brain Axis in Acute and Chronic Brain Diseases,” Current Opinion in Neurobiology 61 (2020): 1–9. 31812830 10.1016/j.conb.2019.11.009

[73] V. Braniste , M. Al‐Asmakh , C. Kowal , et al., “The Gut Microbiota Influences Blood‐Brain Barrier Permeability in Mice,” Science Translational Medicine 6 (2014): 263ra158. 10.1126/scitranslmed.3009759 PMC4396848 25411471

[74] J. Liu , H. Li , T. Gong , et al., “Anti‐Neuroinflammatory Effect of Short‐Chain Fatty Acid Acetate Against Alzheimer's Disease via Upregulating GPR41 and Inhibiting ERK/JNK/NF‐κB,” Journal of Agricultural and Food Chemistry 68 (2020): 7152–7161. 32583667 10.1021/acs.jafc.0c02807

[75] M. E. Caetano‐Silva , L. Rund , N. T. Hutchinson , J. A. Woods , A. J. Steelman , and R. W. Johnson , “Inhibition of Inflammatory Microglia by Dietary Fiber and Short‐Chain Fatty Acids,” Scientific Reports 13 (2023): 2819. 36797287 10.1038/s41598-022-27086-x PMC9935636

[76] M. Datta , O. Staszewski , E. Raschi , et al., “Histone Deacetylases 1 and 2 Regulate Microglia Function During Development, Homeostasis, and Neurodegeneration in a Context‐Dependent Manner,” Immunity 48 (2018): 514–529.e516. 29548672 10.1016/j.immuni.2018.02.016

[77] J. Sun , J. Xu , B. Yang , et al., “Effect of Clostridium Butyricum Against Microglia‐Mediated Neuroinflammation in Alzheimer's Disease via Regulating Gut Microbiota and Metabolites Butyrate,” Molecular Nutrition & Food Research 64 (2020): e1900636. 31835282 10.1002/mnfr.201900636

[78] R. Corrêa‐Oliveira , J. L. Fachi , A. Vieira , F. T. Sato , and M. A. R. Vinolo , “Regulation of Immune Cell Function by Short‐Chain Fatty Acids,” Clinical & Translational Immunology 5 (2016): 5. 10.1038/cti.2016.17 PMC4855267 27195116

[79] P. V. Chang , L. Hao , S. Offermanns , and R. Medzhitov , “The Microbial Metabolite Butyrate Regulates Intestinal Macrophage Function via Histone Deacetylase Inhibition,” Proceedings of the National Academy of Sciences of the United States of America 111 (2014): 2247–2252. 24390544 10.1073/pnas.1322269111 PMC3926023

[80] M. Platten , E. A. A. Nollen , U. F. Röhrig , F. Fallarino , and C. A. Opitz , “Tryptophan Metabolism as a Common Therapeutic Target in Cancer, Neurodegeneration and Beyond,” Nature Reviews. Drug Discovery 18 (2019): 379–401. 30760888 10.1038/s41573-019-0016-5

[81] F. Dong and G. H. Perdew , “The Aryl Hydrocarbon Receptor as a Mediator of Host‐Microbiota Interplay,” Gut Microbes 12, no. 1 (2020): 1–17. 10.1080/19490976.2020.1859812 PMC7781536 33382356

[82] N. Ma , T. He , L. J. Johnston , and X. Ma , “Host‐Microbiome Interactions: The Aryl Hydrocarbon Receptor as a Critical Node in Tryptophan Metabolites to Brain Signaling,” Gut Microbes 11, no. 5 (2020): 1203–1219. 32401136 10.1080/19490976.2020.1758008 PMC7524279

[83] G. Jing , X. Kang , L. Hongnan , et al., “Impact of the Gut Microbiota on Intestinal Immunity Mediated by Tryptophan Metabolism,” Frontiers in Cellular and Infection Microbiology 8 (2018): 13. 29468141 10.3389/fcimb.2018.00013 PMC5808205

[84] A. Agus , J. Planchais , and H. Sokol , “Gut Microbiota Regulation of Tryptophan Metabolism in Health and Disease – ScienceDirect,” Cell Host & Microbe 23, no. 6 (2018): 716–724. 29902437 10.1016/j.chom.2018.05.003

[85] Y. Deng , M. Zhou , J. Wang , J. Yao , and R. Gao , “Involvement of the Microbiota‐Gut‐Brain Axis in Chronic Restraint Stress: Disturbances of the Kynurenine Metabolic Pathway in Both the Gut and Brain,” Gut Microbes 13, no. 1 (2021): 1–16. 10.1080/19490976.2020.1869501 PMC7872056 33535879

[86] Y. H. Kwon , H. Wang , E. Denou , et al., “Modulation of Gut Microbiota Composition by Serotonin Signaling Influences Intestinal Immune Response and Susceptibility to Colitis,” Cellular and Molecular Gastroenterology and Hepatology 7, no. 4 (2019): 709–728. 30716420 10.1016/j.jcmgh.2019.01.004 PMC6462823

[87] C. S. Reigstad , C. E. Salmonson , J. F. R. Iii , et al., “Gut Microbes Promote Colonic Serotonin Production Through an Effect of Short‐Chain Fatty Acids on Enterochromaffin Cells,” FASEB Journal 29, no. 4 (2015): 1395–1403. 25550456 10.1096/fj.14-259598 PMC4396604

[88] Y. H. Kwon , H. Wang , E. Denou , et al., “Modulation of Gut Microbiota Composition by Serotonin Signaling Influences Intestinal Immune Response and Susceptibility to Colitis,” Cellular and Molecular Gastroenterology and Hepatology 7 (2019): 709–728. 30716420 10.1016/j.jcmgh.2019.01.004 PMC6462823

[89] L. M. Giil , O. Midttun , H. Refsum , et al., “Kynurenine Pathway Metabolites in Alzheimer's Disease,” Journal of Alzheimer's Disease 60 (2017): 495–504. 10.3233/JAD-170485 28869479

[90] V. Rothhammer , I. D. Mascanfroni , L. Bunse , et al., “Type I Interferons and Microbial Metabolites of Tryptophan Modulate Astrocyte Activity and Central Nervous System Inflammation via the Aryl Hydrocarbon Receptor,” Nature Medicine 22 (2016): 586–597. 10.1038/nm.4106 PMC4899206 27158906

[91] G. Z. Wei , K. A. Martin , P. Y. Xing , et al., “Tryptophan‐Metabolizing Gut Microbes Regulate Adult Neurogenesis via the Aryl Hydrocarbon Receptor,” Proceedings of the National Academy of Sciences of the United States of America 118 (2021): e2021091118. 34210797 10.1073/pnas.2021091118 PMC8271728

[92] J. Sun , Y. Zhang , Y. Kong , et al., “Microbiota‐Derived Metabolite Indoles Induced Aryl Hydrocarbon Receptor Activation and Inhibited Neuroinflammation in APP/PS1 Mice,” Brain, Behavior, and Immunity 106 (2022): 76–88. 35961580 10.1016/j.bbi.2022.08.003

[93] S. Pan , Y. Zhang , T. Ye , et al., “A High‐Tryptophan Diet Alleviated Cognitive Impairment and Neuroinflammation in APP/PS1 Mice Through Activating Aryl Hydrocarbon Receptor via the Regulation of Gut Microbiota,” Molecular Nutrition & Food Research 68 (2024): e2300601. 38031265 10.1002/mnfr.202300601

[94] A. Agus , J. Planchais , and H. Sokol , “Gut Microbiota Regulation of Tryptophan Metabolism in Health and Disease,” Cell Host & Microbe 23 (2018): 716–724. 29902437 10.1016/j.chom.2018.05.003

[95] Q. Zhang , Y. Sun , Z. He , et al., “Kynurenine Regulates NLRP2 Inflammasome in Astrocytes and Its Implications in Depression,” Brain, Behavior, and Immunity 88 (2020): 471–481. 32283293 10.1016/j.bbi.2020.04.016

[96] W. H. Tang , Z. Wang , B. S. Levison , et al., “Intestinal Microbial Metabolism of Phosphatidylcholine and Cardiovascular Risk,” New England Journal of Medicine 368 (2013): 1575–1584. 23614584 10.1056/NEJMoa1109400 PMC3701945

[97] Y. Zhang , G. Wang , R. Li , et al., “Trimethylamine N‐Oxide Aggravated Cognitive Impairment From APP/PS1 Mice and Protective Roles of Voluntary Exercise,” Neurochemistry International 162 (2023): 105459. 36460238 10.1016/j.neuint.2022.105459

[98] V. E. Brunt , T. J. LaRocca , A. E. Bazzoni , et al., “The Gut Microbiome‐Derived Metabolite Trimethylamine N‐Oxide Modulates Neuroinflammation and Cognitive Function With Aging,” Geroscience 43 (2021): 377–394. 32862276 10.1007/s11357-020-00257-2 PMC8050157

[99] X. Hu , Y. Zhang , C. Gu , et al., “TMAO Promotes Dementia Progression by Mediating the PI3K/Akt/mTOR Pathway,” Tissue & Cell 81 (2023): 102034. 36753814 10.1016/j.tice.2023.102034

[100] Q. Gao , Y. Wang , X. Wang , et al., “Decreased Levels of Circulating Trimethylamine N‐Oxide Alleviate Cognitive and Pathological Deterioration in Transgenic Mice: A Potential Therapeutic Approach for Alzheimer's Disease,” Aging (Albany NY) 11 (2019): 8642–8663. 31612864 10.18632/aging.102352 PMC6814608

[101] S. G. Sorboni , H. S. Moghaddam , R. Jafarzadeh‐Esfehani , and S. Soleimanpour , “A Comprehensive Review on the Role of the Gut Microbiome in Human Neurological Disorders,” Clinical Microbiology Reviews 35 (2022): e0033820. 34985325 10.1128/CMR.00338-20 PMC8729913

[102] G. Vighi , F. Marcucci , L. Sensi , G. Di Cara , and F. Frati , “Allergy and the Gastrointestinal System,” Clinical and Experimental Immunology 153, no. Suppl 1 (2008): 3–6. 18721321 10.1111/j.1365-2249.2008.03713.x PMC2515351

[103] T. C. Fung , C. A. Olson , and E. Y. Hsiao , “Interactions Between the Microbiota, Immune and Nervous Systems in Health and Disease,” Nature Neuroscience 20 (2017): 145–155. 28092661 10.1038/nn.4476 PMC6960010

[104] W. Zhang , D. Xiao , Q. Mao , and H. Xia , “Role of Neuroinflammation in Neurodegeneration Development,” Signal Transduction and Targeted Therapy 8 (2023): 267. 37433768 10.1038/s41392-023-01486-5 PMC10336149

[105] D. Erny and M. Prinz , “How Microbiota Shape Microglial Phenotypes and Epigenetics,” Glia 68 (2020): 1655–1672. 32181523 10.1002/glia.23822

[106] N. Saiyasit , T. Chunchai , D. Prus , et al., “Gut Dysbiosis Develops Before Metabolic Disturbance and Cognitive Decline in High‐Fat Diet‐Induced Obese Condition,” Nutrition 69 (2020): 110576. 31580986 10.1016/j.nut.2019.110576

[107] M. Nguyen and N. W. Palm , “Gut Instincts in Neuroimmunity From the Eighteenth to Twenty‐First Centuries,” Seminars in Immunopathology 44 (2022): 569–579. 35786740 10.1007/s00281-022-00948-2 PMC9519704

[108] J. L. Round and S. K. Mazmanian , “Inducible Foxp3+ Regulatory T‐Cell Development by a Commensal Bacterium of the Intestinal Microbiota,” Proceedings of the National Academy of Sciences of the United States of America 107 (2010): 12204–12209. 20566854 10.1073/pnas.0909122107 PMC2901479

[109] K. Yasuda , Y. Takeuchi , and K. Hirota , “The Pathogenicity of Th17 Cells in Autoimmune Diseases,” Seminars in Immunopathology 41 (2019): 283–297. 30891627 10.1007/s00281-019-00733-8

[110] A. B. Shreiner , J. Y. Kao , and V. B. Young , “The Gut Microbiome in Health and in Disease,” Current Opinion in Gastroenterology 31 (2015): 69–75. 25394236 10.1097/MOG.0000000000000139 PMC4290017

[111] P. M. Smith , M. R. Howitt , N. Panikov , et al., “The Microbial Metabolites, Short‐Chain Fatty Acids, Regulate Colonic Treg Cell Homeostasis,” Science 341 (2013): 569–573. 23828891 10.1126/science.1241165 PMC3807819

[112] Y. J. Liu , B. Tang , F. C. Wang , et al., “Parthenolide Ameliorates Colon Inflammation Through Regulating Treg/Th17 Balance in a Gut Microbiota‐Dependent Manner,” Theranostics 10 (2020): 5225–5241. 32373209 10.7150/thno.43716 PMC7196297

[113] Y. Furusawa , Y. Obata , S. Fukuda , et al., “Commensal Microbe‐Derived Butyrate Induces the Differentiation of Colonic Regulatory T Cells,” Nature 504 (2013): 446–450. 24226770 10.1038/nature12721

[114] C. A. White , E. J. Pone , T. Lam , et al., “Histone Deacetylase Inhibitors Upregulate B Cell microRNAs That Silence AID and Blimp‐1 Expression for Epigenetic Modulation of Antibody and Autoantibody Responses,” Journal of Immunology 193 (2014): 5933–5950. 10.4049/jimmunol.1401702 PMC4258531 25392531

[115] W. Wu , M. Sun , F. Chen , et al., “Microbiota Metabolite Short‐Chain Fatty Acid Acetate Promotes Intestinal IgA Response to Microbiota Which Is Mediated by GPR43,” Mucosal Immunology 10 (2017): 946–956. 27966553 10.1038/mi.2016.114 PMC5471141

[116] K. Lee , S. Hwang , D. J. Paik , W. K. Kim , J. M. Kim , and J. Youn , “Bacillus‐Derived Poly‐γ‐Glutamic Acid Reciprocally Regulates the Differentiation of T Helper 17 and Regulatory T Cells and Attenuates Experimental Autoimmune Encephalomyelitis,” Clinical and Experimental Immunology 170 (2012): 66–76. 22943202 10.1111/j.1365-2249.2012.04637.x PMC3444718

[117] S. Kim , H. Kim , Y. S. Yim , et al., “Maternal Gut Bacteria Promote Neurodevelopmental Abnormalities in Mouse Offspring,” Nature 549 (2017): 528–532. 28902840 10.1038/nature23910 PMC5870873

[118] E. Kim , D. Paik , R. N. Ramirez , et al., “Maternal Gut Bacteria Drive Intestinal Inflammation in Offspring With Neurodevelopmental Disorders by Altering the Chromatin Landscape of CD4(+) T Cells,” Immunity 55 (2022): 145–158.e147. 34879222 10.1016/j.immuni.2021.11.005 PMC8755621

[119] O. L. Rojas , A. K. Pröbstel , E. A. Porfilio , et al., “Recirculating Intestinal IgA‐Producing Cells Regulate Neuroinflammation via IL‐10,” Cell 176 (2019): 610–624.e618. 30612739 10.1016/j.cell.2018.11.035 PMC6903689

[120] O. Pabst and E. Slack , “IgA and the Intestinal Microbiota: The Importance of Being Specific,” Mucosal Immunology 13 (2020): 12–21. 31740744 10.1038/s41385-019-0227-4 PMC6914667

[121] E. E. Alexeev , J. M. Lanis , D. J. Kao , et al., “Microbiota‐Derived Indole Metabolites Promote Human and Murine Intestinal Homeostasis Through Regulation of Interleukin‐10 Receptor,” American Journal of Pathology 188 (2018): 1183–1194. 29454749 10.1016/j.ajpath.2018.01.011 PMC5906738

[122] G. Clarke , R. M. Stilling , P. J. Kennedy , C. Stanton , J. F. Cryan , and T. G. Dinan , “Minireview: Gut Microbiota: The Neglected Endocrine Organ,” Molecular Endocrinology 28 (2014): 1221–1238. 24892638 10.1210/me.2014-1108 PMC5414803

[123] J. Correale , R. Hohlfeld , and S. E. Baranzini , “The Role of the Gut Microbiota in Multiple Sclerosis,” Nature Reviews. Neurology 18 (2022): 544–558. 35931825 10.1038/s41582-022-00697-8

[124] J. Ma , R. Wang , Y. Chen , Z. Wang , and Y. Dong , “5‐HT Attenuates Chronic Stress‐Induced Cognitive Impairment in Mice Through Intestinal Flora Disruption,” Journal of Neuroinflammation 20 (2023): 23. 36737776 10.1186/s12974-023-02693-1 PMC9896737

[125] J. Lu , C. Zhang , J. Lv , et al., “Antiallergic Drug Desloratadine as a Selective Antagonist of 5HT(2A) Receptor Ameliorates Pathology of Alzheimer's Disease Model Mice by Improving Microglial Dysfunction,” Aging Cell 20 (2021): e13286. 33369003 10.1111/acel.13286 PMC7811850

[126] M. Wang , H. F. Zong , K. W. Chang , et al., “5‐HT(1A)R Alleviates Aβ‐Induced Cognitive Decline and Neuroinflammation Through Crosstalk With NF‐κB Pathway in Mice,” International Immunopharmacology 82 (2020): 106354. 32143008 10.1016/j.intimp.2020.106354

[127] P. Zhu , T. Lu , J. Wu , et al., “Gut Microbiota Drives Macrophage‐Dependent Self‐Renewal of Intestinal Stem Cells via Niche Enteric Serotonergic Neurons,” Cell Research 32 (2022): 555–569. 35379903 10.1038/s41422-022-00645-7 PMC9160288

[128] M. Wan , L. Ding , D. Wang , J. Han , and P. Gao , “Serotonin: A Potent Immune Cell Modulator in Autoimmune Diseases,” Frontiers in Immunology 11 (2020): 186. 32117308 10.3389/fimmu.2020.00186 PMC7026253

[129] K. Glebov , M. Löchner , R. Jabs , et al., “Serotonin Stimulates Secretion of Exosomes From Microglia Cells,” Glia 63 (2015): 626–634. 25451814 10.1002/glia.22772

[130] J. Lloyd‐Price , C. Arze , A. N. Ananthakrishnan , et al., “Multi‐Omics of the Gut Microbial Ecosystem in Inflammatory Bowel Diseases,” Nature 569 (2019): 655–662. 31142855 10.1038/s41586-019-1237-9 PMC6650278

[131] J. Y. Lee , J. A. Hall , L. Kroehling , et al., “Serum Amyloid A Proteins Induce Pathogenic Th17 Cells and Promote Inflammatory Disease,” Cell 180 (2020): 79–91.e16. 31866067 10.1016/j.cell.2019.11.026 PMC7039443

[132] Y. Yu , J. Liu , S. Q. Li , L. Peng , and R. D. Ye , “Serum Amyloid a Differentially Activates Microglia and Astrocytes via the PI3K Pathway,” Journal of Alzheimer's Disease 38 (2014): 133–144. 10.3233/JAD-130818 23948927

[133] A. Lin , J. Liu , P. Gong , et al., “Serum Amyloid A Inhibits Astrocyte Migration via Activating p38 MAPK,” Journal of Neuroinflammation 17 (2020): 254. 32861245 10.1186/s12974-020-01924-z PMC7456509

[134] J. Zhang , K. F. Ke , Z. Liu , Y. H. Qiu , and Y. P. Peng , “Th17 Cell‐Mediated Neuroinflammation Is Involved in Neurodegeneration of abeta1‐42‐Induced Alzheimer's Disease Model Rats,” PLoS One 8 (2013): e75786. 24124514 10.1371/journal.pone.0075786 PMC3790825

[135] M. A. Erickson and A. P. Mahankali , “Interactions of Serum Amyloid A Proteins With the Blood‐Brain Barrier: Implications for Central Nervous System Disease,” International Journal of Molecular Sciences 25, no. 12 (2024): 6607. 38928312 10.3390/ijms25126607 PMC11204325

[136] Y. Han , B. Wang , H. Gao , et al., “Vagus Nerve and Underlying Impact on the Gut Microbiota‐Brain Axis in Behavior and Neurodegenerative Diseases,” Journal of Inflammation Research 15 (2022): 6213–6230. 36386584 10.2147/JIR.S384949 PMC9656367

[137] B. Bonaz , V. Sinniger , and S. Pellissier , “The Vagus Nerve in the Neuro‐Immune Axis: Implications in the Pathology of the Gastrointestinal Tract,” Frontiers in Immunology 8 (2017): 1452. 29163522 10.3389/fimmu.2017.01452 PMC5673632

[138] B. O. Schroeder and F. Bäckhed , “Signals From the Gut Microbiota to Distant Organs in Physiology and Disease,” Nature Medicine 22 (2016): 1079–1089. 10.1038/nm.4185 27711063

[139] M. Yesiltepe , B. Cimen , and Y. Sara , “Effects of Chronic Vagal Nerve Stimulation in the Treatment of Beta‐Amyloid‐Induced Neuropsychiatric Symptoms,” European Journal of Pharmacology 931 (2022): 175179. 35973478 10.1016/j.ejphar.2022.175179

[140] L. Bonfili , V. Cecarini , S. Berardi , et al., “Microbiota Modulation Counteracts Alzheimer's Disease Progression Influencing Neuronal Proteolysis and Gut Hormones Plasma Levels,” Scientific Reports 7 (2017): 2426. 28546539 10.1038/s41598-017-02587-2 PMC5445077

[141] Y. Kobayashi , H. Sugahara , K. Shimada , et al., “Therapeutic Potential of Bifidobacterium Breve Strain A1 for Preventing Cognitive Impairment in Alzheimer's Disease,” Scientific Reports 7 (2017): 13510. 29044140 10.1038/s41598-017-13368-2 PMC5647431

[142] B. Bonaz , T. Bazin , and S. Pellissier , “The Vagus Nerve at the Interface of the Microbiota‐Gut‐Brain Axis,” Frontiers in Neuroscience 12 (2018): 49. 29467611 10.3389/fnins.2018.00049 PMC5808284

[143] G. Agirman , K. B. Yu , and E. Y. Hsiao , “Signaling Inflammation Across the Gut‐Brain Axis,” Science 374 (2021): 1087–1092. 34822299 10.1126/science.abi6087

[144] S. C. Payne , J. B. Furness , O. Burns , et al., “Anti‐Inflammatory Effects of Abdominal Vagus Nerve Stimulation on Experimental Intestinal Inflammation,” Frontiers in Neuroscience 13 (2019): 418. 31133776 10.3389/fnins.2019.00418 PMC6517481

[145] F. A. Pinho‐Ribeiro , W. A. Verri, Jr. , and I. M. Chiu , “Nociceptor Sensory Neuron‐Immune Interactions in Pain and Inflammation,” Trends in Immunology 38 (2017): 5–19. 27793571 10.1016/j.it.2016.10.001 PMC5205568

[146] H. Wang , M. Yu , M. Ochani , et al., “Nicotinic Acetylcholine Receptor alpha7 Subunit Is an Essential Regulator of Inflammation,” Nature 421 (2003): 384–388. 12508119 10.1038/nature01339

[147] C. Pellegrini , L. Antonioli , R. Colucci , C. Blandizzi , and M. Fornai , “Interplay Among Gut Microbiota, Intestinal Mucosal Barrier and Enteric Neuro‐Immune System: A Common Path to Neurodegenerative Diseases?,” Acta Neuropathologica 136 (2018): 345–361. 29797112 10.1007/s00401-018-1856-5

[148] B. Obermeier , R. Daneman , and R. M. Ransohoff , “Development, Maintenance and Disruption of the Blood‐Brain Barrier,” Nature Medicine 19 (2013): 1584–1596. 10.1038/nm.3407 PMC4080800 24309662

[149] N. Kurita , K. Yamashiro , T. Kuroki , et al., “Metabolic Endotoxemia Promotes Neuroinflammation After Focal Cerebral Ischemia,” Journal of Cerebral Blood Flow and Metabolism 40 (2020): 2505–2520. 31910709 10.1177/0271678X19899577 PMC7820690

[150] M. S. Desai , A. M. Seekatz , N. M. Koropatkin , et al., “A Dietary Fiber‐Deprived Gut Microbiota Degrades the Colonic Mucus Barrier and Enhances Pathogen Susceptibility,” Cell 167 (2016): 1339–1353.e1321. 27863247 10.1016/j.cell.2016.10.043 PMC5131798

[151] Y. Mou , Y. Du , L. Zhou , et al., “Gut Microbiota Interact With the Brain Through Systemic Chronic Inflammation: Implications on Neuroinflammation, Neurodegeneration, and Aging,” Frontiers in Immunology 13 (2022): 796288. 35464431 10.3389/fimmu.2022.796288 PMC9021448

[152] W. J. Lukiw , “Bacteroides Fragilis Lipopolysaccharide and Inflammatory Signaling in Alzheimer's Disease,” Frontiers in Microbiology 7 (2016): 1544. 27725817 10.3389/fmicb.2016.01544 PMC5035737

[153] C. R. A. Batista , G. F. Gomes , E. Candelario‐Jalil , B. L. Fiebich , and A. C. P. de Oliveira , “Lipopolysaccharide‐Induced Neuroinflammation as a Bridge to Understand Neurodegeneration,” International Journal of Molecular Sciences 20, no. 9 (2019): 2293. 31075861 10.3390/ijms20092293 PMC6539529

[154] V. M. Choi , J. Herrou , A. L. Hecht , et al., “Activation of Bacteroides Fragilis Toxin by a Novel Bacterial Protease Contributes to Anaerobic Sepsis in Mice,” Nature Medicine 22 (2016): 563–567. 10.1038/nm.4077 PMC4860040 27089515

[155] Y. Belkaid and T. W. Hand , “Role of the Microbiota in Immunity and Inflammation,” Cell 157 (2014): 121–141. 24679531 10.1016/j.cell.2014.03.011 PMC4056765

[156] Y. Zhao , V. Jaber , and W. J. Lukiw , “Secretory Products of the Human GI Tract Microbiome and Their Potential Impact on Alzheimer's Disease (AD): Detection of Lipopolysaccharide (LPS) in AD Hippocampus,” Frontiers in Cellular and Infection Microbiology 7 (2017): 318. 28744452 10.3389/fcimb.2017.00318 PMC5504724

[157] A. Bachem , C. Makhlouf , K. J. Binger , et al., “Microbiota‐Derived Short‐Chain Fatty Acids Promote the Memory Potential of Antigen‐Activated CD8(+) T Cells,” Immunity 51 (2019): 285–297.e285. 31272808 10.1016/j.immuni.2019.06.002

[158] Y. Zhao , L. Cong , V. Jaber , and W. J. Lukiw , “Microbiome‐Derived Lipopolysaccharide Enriched in the Perinuclear Region of Alzheimer's Disease Brain,” Frontiers in Immunology 8 (2017): 1064. 28928740 10.3389/fimmu.2017.01064 PMC5591429

[159] Y. Zhao and W. J. Lukiw , “Microbiome‐Generated Amyloid and Potential Impact on Amyloidogenesis in Alzheimer's Disease (AD),” Journal of Nature and Science 1, no. 7 (2015): e138. PMC4469284 26097896

[160] S. H. Rhee , “Lipopolysaccharide: Basic Biochemistry, Intracellular Signaling, and Physiological Impacts in the Gut,” Intestinal Research 12 (2014): 90–95. 25349574 10.5217/ir.2014.12.2.90 PMC4204704

[161] M. A. Daulatzai , “Chronic Functional Bowel Syndrome Enhances Gut‐Brain Axis Dysfunction, Neuroinflammation, Cognitive Impairment, and Vulnerability to Dementia,” Neurochemical Research 39 (2014): 624–644. 24590859 10.1007/s11064-014-1266-6

[162] Y. Zhao , P. Dua , and W. J. Lukiw , “Microbial Sources of Amyloid and Relevance to Amyloidogenesis and Alzheimer's Disease (AD),” Journal of Alzheimer Disease & Parkinsonism 5 (2015): 177. 10.4172/2161-0460.1000177 PMC4428612 25977840

[163] M. S. Khan , M. Ikram , J. S. Park , T. J. Park , and M. O. Kim , “Gut Microbiota, Its Role in Induction of Alzheimer's Disease Pathology, and Possible Therapeutic Interventions: Special Focus on Anthocyanins,” Cells 9 (2020): 853. 32244729 10.3390/cells9040853 PMC7226756

[164] J. Kim , H. J. Lee , S. K. Park , et al., “Donepezil Regulates LPS and Aβ‐Stimulated Neuroinflammation Through MAPK/NLRP3 Inflammasome/STAT3 Signaling,” International Journal of Molecular Sciences 22 (2021): 10637. 34638977 10.3390/ijms221910637 PMC8508964

[165] T. X. Yang , Y. F. Zhu , C. C. Wang , et al., “EPA‐Enriched Plasmalogen Attenuates the Cytotoxic Effects of LPS‐Stimulated Microglia on the SH‐SY5Y Neuronal Cell Line,” Brain Research Bulletin 186 (2022): 143–152. 35728742 10.1016/j.brainresbull.2022.06.002

[166] Y. Zhao , L. Cong , and W. J. Lukiw , “Lipopolysaccharide (LPS) Accumulates in Neocortical Neurons of Alzheimer's Disease (AD) Brain and Impairs Transcription in Human Neuronal‐Glial Primary co‐Cultures,” Frontiers in Aging Neuroscience 9 (2017): 407. 29311897 10.3389/fnagi.2017.00407 PMC5732913

[167] P. Kesika , N. Suganthy , B. S. Sivamaruthi , and C. Chaiyasut , “Role of Gut‐Brain Axis, Gut Microbial Composition, and Probiotic Intervention in Alzheimer's Disease,” Life Sciences 264 (2021): 118627. 33169684 10.1016/j.lfs.2020.118627

[168] J. T. Matheson and R. M. D. Holsinger , “The Role of Fecal Microbiota Transplantation in the Treatment of Neurodegenerative Diseases: A Review,” International Journal of Molecular Sciences 24 (2023): 1001. 36674517 10.3390/ijms24021001 PMC9864694

[169] X. Wang , G. Sun , T. Feng , et al., “Sodium Oligomannate Therapeutically Remodels Gut Microbiota and Suppresses Gut Bacterial Amino Acids‐Shaped Neuroinflammation to Inhibit Alzheimer's Disease Progression,” Cell Research 29 (2019): 787–803. 31488882 10.1038/s41422-019-0216-x PMC6796854

[170] M. S. Kim , Y. Kim , H. Choi , et al., “Transfer of a Healthy Microbiota Reduces Amyloid and Tau Pathology in an Alzheimer's Disease Animal Model,” Gut 69 (2020): 283–294. 31471351 10.1136/gutjnl-2018-317431

[171] S. H. Park , J. H. Lee , J. Shin , et al., “Cognitive Function Improvement After Fecal Microbiota Transplantation in Alzheimer's Dementia Patient: A Case Report,” Current Medical Research and Opinion 37 (2021): 1739–1744. 34289768 10.1080/03007995.2021.1957807

[172] M. R. Minter , C. Zhang , V. Leone , et al., “Antibiotic‐Induced Perturbations in Gut Microbial Diversity Influences Neuro‐Inflammation and Amyloidosis in a Murine Model of Alzheimer's Disease,” Scientific Reports 6 (2016): 30028. 27443609 10.1038/srep30028 PMC4956742

[173] J. D. Hoffman , L. M. Yanckello , G. Chlipala , et al., “Dietary Inulin Alters the Gut Microbiome, Enhances Systemic Metabolism and Reduces Neuroinflammation in an APOE4 Mouse Model,” PLoS One 14 (2019): e0221828. 31461505 10.1371/journal.pone.0221828 PMC6713395

[174] D. Wang , L. Ho , J. Faith , et al., “Role of Intestinal Microbiota in the Generation of Polyphenol‐Derived Phenolic Acid Mediated Attenuation of Alzheimer's Disease β‐Amyloid Oligomerization,” Molecular Nutrition & Food Research 59 (2015): 1025–1040. 25689033 10.1002/mnfr.201400544 PMC4498582

[175] C. S. Kim , L. Cha , M. Sim , et al., “Probiotic Supplementation Improves Cognitive Function and Mood With Changes in Gut Microbiota in Community‐Dwelling Older Adults: A Randomized, Double‐Blind, Placebo‐Controlled, Multicenter Trial,” Journals of Gerontology. Series A, Biological Sciences and Medical Sciences 76 (2021): 32–40. 32300799 10.1093/gerona/glaa090 PMC7861012

[176] V. Cecarini , L. Bonfili , O. Gogoi , et al., “Neuroprotective Effects of p62(SQSTM1)‐engineered Lactic Acid Bacteria in Alzheimer's Disease: A Pre‐Clinical Study,” Aging (Albany NY) 12 (2020): 15995–16020. 32855357 10.18632/aging.103900 PMC7485699

[177] H. Kaur , K. Nagamoto‐Combs , S. Golovko , M. Y. Golovko , M. G. Klug , and C. K. Combs , “Probiotics Ameliorate Intestinal Pathophysiology in a Mouse Model of Alzheimer's Disease,” Neurobiology of Aging 92 (2020): 114–134. 32417748 10.1016/j.neurobiolaging.2020.04.009 PMC7269849

[178] A. Labarre , E. Guitard , G. Tossing , et al., “Fatty Acids Derived From the Probiotic Lacticaseibacillus Rhamnosus HA‐114 Suppress Age‐Dependent Neurodegeneration,” Communications Biology 5 (2022): 1340. 36477191 10.1038/s42003-022-04295-8 PMC9729297

[179] P. Sittipo , J. Choi , S. Lee , and Y. K. Lee , “The Function of Gut Microbiota in Immune‐Related Neurological Disorders: A Review,” Journal of Neuroinflammation 19 (2022): 154. 35706008 10.1186/s12974-022-02510-1 PMC9199126

[180] K. Suganya and B. S. Koo , “Gut‐Brain Axis: Role of Gut Microbiota on Neurological Disorders and How Probiotics/Prebiotics Beneficially Modulate Microbial and Immune Pathways to Improve Brain Functions,” International Journal of Molecular Sciences 21 (2020): 7551. 33066156 10.3390/ijms21207551 PMC7589356

[181] M. M. Kendall and V. Sperandio , “Gut Microbes Regroup to Aid Defence After Infection,” Nature 592 (2021): 29–31. 34728860 10.1038/d41586-021-00642-7 PMC8162939

[182] I. Martínez , J. C. Stegen , M. X. Maldonado‐Gómez , et al., “The Gut Microbiota of Rural Papua New Guineans: Composition, Diversity Patterns, and Ecological Processes,” Cell Reports 11 (2015): 527–538. 25892234 10.1016/j.celrep.2015.03.049

[183] D. O. Seo and D. M. Holtzman , “Current Understanding of the Alzheimer's Disease‐Associated Microbiome and Therapeutic Strategies,” Experimental & Molecular Medicine 56, no. 1 (2024): 86–94. 38172602 10.1038/s12276-023-01146-2 PMC10834451

